# DNA methylation dynamics during the interaction of wheat progenitor *Aegilops tauschii* with the obligate biotrophic fungus *Blumeria graminis* f. sp. *tritici*

**DOI:** 10.1111/nph.15432

**Published:** 2018-09-06

**Authors:** Shuaifeng Geng, Xingchen Kong, Gaoyuan Song, Meiling Jia, Jiantao Guan, Fang Wang, Zhengrui Qin, Liang Wu, Xiujin Lan, Aili Li, Long Mao

**Affiliations:** ^1^ National Key Facility for Crop Gene Resources and Genetic Improvement Institute of Crop Science Chinese Academy of Agricultural Sciences Beijing 100081 China; ^2^ Triticeae Research Institute Sichuan Agricultural University Chengdu Sichuan 611130 China; ^3^ Department of Agronomy College of Agriculture and Biotechnology Zhejiang University Hangzhou 310058 China

**Keywords:** *Aegilops tauschii*, AGO4a, *Blumeria graminis* f. sp. *tritici* (*Bgt*), DNA methylation, siRNA, wheat

## Abstract

- DNA methylation is dynamically involved in plant immunity, but little information is known about its roles in plant interactions with biotrophic fungi, especially in temperate grasses such as wheat (*Triticum aestivum*).
- Using wheat diploid progenitor *Aegilops tauschii* accession AL8/78, the genome of which has been sequenced, we assessed the extent of DNA methylation in response to infection with *Blumeria graminis* f. sp. *tritici* (*Bgt*), which causes powdery mildew.
- Upon *Bgt* infection, *ARGONAUTE4a* (*AGO4a*) was significantly downregulated in *A. tauschii*, which was accompanied by a substantial reduction in AGO4a‐sorted 24‐nt siRNA levels, especially for genes near transposable elements (TAGs). Bisulfite sequencing revealed abundant differentially methylated regions (DMRs) with CHH hypomethylation. TAGs bearing CHH‐hypomethylated DMRs were enriched for ‘response to stress’ functions, including receptor kinase, peroxidase, and pathogenesis‐related genes. Virus‐induced gene silencing (VIGS) of a *DOMAINS REARRANGED METHYLASE 2* (*DRM2*) homolog enhanced plant resistance to *Bgt*. The effect of CHH hypomethylation was exemplified by the upregulation of a pathogenesis‐related β‐1,3‐glucanse gene implicated in *Bgt* defense.
- These findings support the idea that dynamic DNA methylation represents a regulatory layer in the complex mechanism of plant immunity, which could be exploited to improve disease resistance in common wheat.

## Introduction

Powdery mildew caused by *Blumeria graminis* f. sp. *tritici* (*Bgt*) is one of the most destructive fungal pathogens of common wheat (*Triticum aestivum*) worldwide (Bourras *et al*., 2015; Parlange *et al*., 2015). Many wheat varieties exhibit race‐specific resistance, which is easily overcome by new *Bgt* races. Therefore, understanding the molecular mechanisms of *Bgt* infection in wheat is pivotal for producing varieties with durable resistance to powdery mildew. Plants protect themselves from pathogen attack through pathogen‐associated molecular pattern (PAMP)‐triggered immunity (PTI) and effector‐triggered immunity (ETI) (Jones & Dangl, 2006; Maekawa *et al*., 2011). The activation of both PTI and ETI causes massive global transcriptional reprogramming, which is strictly controlled by various regulatory mechanisms, including epigenetic regulation (Pandey & Somssich, 2009; Rushton *et al*., 2010).

DNA methylation is an important epigenetic regulatory mechanism involving the addition of a methyl group to cytosines in three sequence contexts: the symmetric CG and CHG and the asymmetric CHH contexts, where H is any nucleotide except G (Cokus *et al*., 2008; Lister *et al*., 2008). DNA methylation is required for transcriptional gene silencing (TGS) and for maintaining genome integrity by silencing transposable elements (TEs). *De novo* methylation in all sequence contexts is mediated by the small RNA‐directed DNA methylation (RdDM) pathway (Matzke & Mosher, 2014). This pathway also stabilizes the methylated sites where 24‐nt siRNAs are loaded into the ARGONAUTE 4 protein (AGO4) to complement RNA polymerase V transcripts, which recruit the methylase DRM2 to carry out DNA methylation reactions (Matzke & Mosher, 2014; Deleris *et al*., 2016). Three types of methylases, METHYLTRANSFERASE 1 (MET1), chromomethylases CMT2/3, and DRM2, are responsible for CG, CHG, and CHH methylation, respectively (Lindroth *et al*., 2001; Cao & Jacobsen, 2002; Kankel *et al*., 2003; Law & Jacobsen, 2010). MET1 and CMT3 are often used to maintain CG and CHG methylation, respectively, which is frequently present in long TEs or heterochromatin regions in the plant genome. By contrast, the RdDM pathway (DRM2) maintains the methylation of CHH loci, which are often present near genes and marked by H3K9me2 (Law *et al*., 2013). The mutation of rice (*Oryza sativa*) *OsDRM2* causes an almost complete loss of CHH methylation and reactivates certain small TE‐associated genes (TAGs) (Tan *et al*., 2016).

Recent studies of plant immunity have revealed the expanding roles of epigenetic control, particularly DNA methylation. In *Arabidopsis thaliana*, for example, enhanced defense responses to *Pseudomonas syringae* pv. *tomato* (*Pst*) DC3000 were observed when genes involved in DNA methylation and demethylation were mutated (Dowen *et al*., 2012; Yu *et al*., 2013; Le *et al*., 2014). Moreover, DNA methylation mediated by 21‐nt siRNAs (Dowen *et al*., 2012) may represent a different mechanism from the RdDM pathway, in which 24‐nt siRNAs play a major role. In rice, treatment with the DNA demethylating agent 5‐azadeoxycytidine enhances resistance to the bacterium *Xanthomonas oryzae* (Akimoto *et al*., 2007), and epigenetic regulation of antagonistic receptors confers rice blast resistance with yield balance (Deng *et al*., 2017). By contrast, resistance to *Pst* DC3000 is compromised in the Arabidopsis DNA methylase mutant, *ros1‐4*. Pathogens have also developed a counter‐defense system against the host plant. For instance, treatment with flg22, a PAMP, inhibits TGS by de‐repressing RdDM targets. These observations suggest that reduced DNA methylation promotes host defense responses against pathogen infection (Yu *et al*., 2013). Among the key components of the RdDM pathway, AGO4a is required for resistance to *P. syringae* in Arabidopsis (Agorio & Vera, 2007); small RNAs (sRNAs) also play a role in plant immunity in this system (Weiberg & Jin, 2015). However, little information is known about DNA methylation dynamics in crops such as wheat, one of the most important staple foods worldwide.

Common wheat is hexaploid, with a high percentage of TE‐derived repetitive sequences and three homoeologous subgenomes: A, B, and D (Choulet *et al*., 2014). Despite the economic importance of wheat, little information is known about the epigenetic regulation of the *Bgt* defense response in wheat. *Aegilops tauschii* is a diploid donor species of wheat that represents a more economically amenable and biologically simple system for studying the regulatory mechanisms of disease resistance than common wheat. Indeed, many *Bgt* races that infect wheat can also infect *A. tauschii*, and resistance resources from *A. tauschii* have been introduced into common wheat via wide hybridization, suggesting that these two related species share similar defense systems (Schneider *et al*., 2008).

In this study, we analyzed the expression patterns of *AGO4a* and 24‐nt small interfering RNAs (siRNAs) associated with this protein. We then sequenced the methylome in a compatible *A. tauschii* accession before and after *Bgt* infection. We found that the decrease in DNA methylation was accompanied by enhanced *Bgt* defense, supporting a role for dynamic DNA methylation in *A. tauschii* immunity. Our study proposes an epigenetic mechanism for antifungal defense involving the regulation of DNA methylation, particularly CHH methylation, in a close relative of common wheat that could be exploited to enhance its resistance to powdery mildew.

## Materials and Methods

### Plant materials and *Bgt* treatment

*A. tauschii* accessions AL8/78 and Y2280 and common wheat PmAm6/Beijing837 BC5F3 lines 1923 and 1930 were kindly provided by Professor Jizeng Jia, Institute of Crop Sciences, CAAS. The plants were grown in a growth chamber at 22°C with a 16 h : 8 h, light : dark cycle (60 μmol m^−2^ s^−1^ photon flux density). For *Bgt* inoculation, *Bgt* conidia were removed from heavily diseased plants by shaking them over a settling tower onto 2‐wk‐old seedlings grown in soil. For sRNA sequencing, the seedlings were harvested at 0 h and 12 h after inoculation (hai). For reverse transcription polymerase chain reaction (RT‐PCR) and methylation level analysis, leaves were also collected at 6, 12, 24 and 48 hai from mock‐treated and inoculated plants. The samples were stored at −80°C before extraction. Am6 is a synthetic amphiploid derived from a cross between *Triticum durum* (AABB) accession DR147 and *A. tauschii* accession Ae39 (Zhou *et al*., 2005). Line 1923 is susceptible and line 1930 is resistant to *Bgt* race no. 15 with an E09 virulence type, a common race in the Beijing area.

### RNA preparation, sequencing, and expression analysis

Total RNA was extracted using TRIzol reagent (Invitrogen). sRNAs were further isolated by resolving the total RNA on a 15% denaturing PAGE gel, and visualized by SYBR Gold (Invitrogen) staining. Gel slices containing 18–28 nt products were excised and the RNA was eluted, purified, and used to construct sRNA libraries. For sRNA sequencing, one library per sample was sequenced on the Illumina HiSeq 2000 platform as described (Mi *et al*., 2008; Wu *et al*., 2010). For quantitative RT‐PCR (qRT‐PCR), total RNA was reverse transcribed with M‐MLV (Promega) using oligo(dT). The cDNAs were used as templates for quantitative RT‐PCR (qRT‐PCR) on an ABI Prism 7300 (Applied Biosystems, Foster City, CA, USA) with SYBR Green as a fluorescent reporter. The number of transcripts was normalized to the mRNA level of the glyceraldehyde‐3‐phosphate dehydrogenase gene (*GAPDH*), which is constitutively expressed in wheat (Li *et al*., 2005; Hong *et al*., 2008). The primers used for PCRs are listed in Supporting Information Table S1.

### Purification of *A. tauschii* AGO4 complexes and associated sRNA

Synthetic peptides AGO4aN (MESHSDDLPPPPC) and AGO4bN (MDPHDGEPAADEC) were used to raise rabbit polyclonal antibodies against the corresponding AGO4 proteins as described by Wu *et al*. (2010). The antisera were immuno‐affinity purified and used for immuno‐precipitation (1 : 50 dilution). The *A. tauschii* AGO4 complexes were immuno‐purified from 2‐wk‐old seedlings as described (Qi *et al*., 2005; Wu *et al*., 2010). The purity of the complexes was examined by SDS‐PAGE, followed by silver staining and immunoblot analysis, and bands of the expected size were confirmed as AGO4 proteins by mass spectrometry. sRNA was then isolated from purified AGO4 complexes using TRIzol reagent (Invitrogen) and sequenced as described above.

### sRNA analysis

sRNA sequence data have been deposited in the Genome Sequence Archive (GSA) (http://bigd.big.ac.cn/gsa/) under accession numbers CRA000896 (total sRNAs) and CRA000897 (AGO4a pull‐down sRNAs). Short reads were parsed to remove 3′ adapters and mapped to the *A. tauschii* genome (ftp://climb.genomics.cn/pub/10.5524/100001_101000/100054/D/Assembly/) (Jia *et al*., 2013). To reduce ambiguity, only perfectly matched reads were used for further analysis. Sequences from chloroplast, mitochondrial, and structural noncoding RNAs, including ribosomal RNAs, transfer RNAs, snoRNAs, and snRNAs, were excluded from the analysis. sRNA reads were normalized by library size and the number of hits to the genome. Protein‐coding genes with adjacent TEs were identified by overlapping the genomic coordinates of TEs and TE fragments with those of protein‐coding genes. sRNAs with perfect genomic matches were used for further analysis. Statistical analysis of the distribution of sRNAs was performed using Wilcoxon paired ranks sum test.

### Construction and sequencing of Methyl‐Seq libraries

For whole‐genome Methyl‐Seq, total genomic DNA was extracted from the leaves of 2‐wk‐old seedlings after 12‐h mock and 12‐h *Bgt* treatment using a Plant Genomic DNA Kit (Tiangen, Beijing, China). Five micrograms of genomic DNA spiked with 26 ng λDNA was fragmented by sonication to a size of 200–300 bp with a Covaris S220, followed by end repair and adenylation. Cytosine‐methylated barcodes were ligated to sonicated DNA as per the manufacturer's instructions. These DNA fragments were treated twice with bisulfite using an EZ DNA Methylation‐Gold™ Kit (Zymo Research, Alameda, CA, USA). The resulting single‐stranded DNA fragments were PCR amplified using KAPA HiFi HotStart Uracil + ReadyMix (2×). The index‐coded samples were clustered on a cBot Cluster Generation System using a TruSeq PE Cluster Kit v3‐cBot‐HS (Illumina) according to the manufacturer's instructions. After cluster generation, one library per sample was prepared and sequenced on the Illumina HiSeq 2500 platform at the Novogene Bioinformatics Institute, and 100‐bp pair‐end reads were generated.

To verify the methylation status of a particular DNA fragment, bisulfite‐treated DNA was subjected to PCR amplification using PCR primers designed using web‐based Kismeth software (Gruntman *et al*., 2008) (Table S1). Amplified PCR fragments were cloned into the pGEM‐T easy vector (Promega) and sequenced. Sequencing data were analyzed using web‐based Kismeth software (Gruntman *et al*., 2008). The Methyl‐Seq sequence data have been deposited in the Genome Sequence Archive (GSA) (http://bigd.big.ac.cn/gsa/) under the accession number CRA000898 (Methyl‐Seq).

### Methyl‐Seq data analysis

Bisulfite‐converted reads were aligned to the wheat reference genome (Ensemble_IWGSP2.25) using Bismark software (v.0.12.5) with default parameters. The conversion rate of cytosine positions was calculated using the Bismark methylation_extractor output.

Differentially methylated regions (DMRs) were identified following a published method (Stroud *et al*., 2013). In brief, the genome was split into continuous 100‐bp windows. Cytosines (Cs) or thymines (Ts) were counted separately in each window for three nucleotide contexts (CG, CHG, or CHH). Windows with at least four Cs with each C covered by at least four reads were selected for DMR analysis. The methylation level for a window was determined as follows: methylation level = Σai/(Σai + bi), where ai is the number of Cs and bi is the number of Ts mapping to the ith C site. The methylation level in each window in the mock was then compared with the corresponding window in the *Bgt*‐treated sample. A methylation difference of 0.4, 0.2 and 0.1 for CG, CHG and CHH, respectively and an adjusted *P*‐value (FDR) of ≤ 0.01 (Fisher's exact test) were used as the cutoff for defining DMRs. DMRs located within 100 bp of each other were merged.

Methylation profiles for metagenes were generated by combining the profiles in three regions: 2 kb upstream of the start codon, the gene body, and 2 kb downstream of the stop codon. Methylated gene bodies consisted of concatenated exons only. For each region, sequences from all genes were divided into 100 bins, and the average methylation level of each bin across each region was calculated and plotted.

### Gene ontology enrichment analysis and MapMan classification

Gene ontology (GO) enrichment analysis was performed using agriGO, a web‐based tool and database for GO analysis (http://bioinfo.cau.edu.cn/agriGO/). GO terms with a corrected FDR of < 0.05 were considered significantly enriched. MapMan (http://mapman.gabipd.org/) was used to identify processes and pathways of specific gene sets (Sreenivasulu *et al*., 2008).

### Virus‐induced gene silencing of barley stripe mosaic virus

The plasmids used in the Virus‐induced gene silencing (VIGS) experiments were based on previously described constructs (Holzberg *et al*., 2002). The *DRM2* and *DRM3* fragments were amplified from cDNA. *Nhe*I restriction sites were used to clone the fragment into the pSS031‐1 vector. Capped transcripts were prepared from three linearized plasmids containing the tripartite barley stripe mosaic virus (BSMV) genome using a mMessage mMachine T7 *in vitro* Transcription Kit (Invitrogen, AM1344), typically resulting in a final concentration of 1–1.5 mg ml^−1^ RNA. *Aegilops tauschii* plants were infected with BSMV RNA following a published protocol (Holzberg *et al*., 2002; Scofield *et al*., 2005).

To estimate *Bgt* infection efficiency, *A. tauschii* leaves (3 cm) were placed onto the surface of 0.5% agarose containing 50 mg l^−1^ 2‐[(4‐chlorophenenyl) methyl]‐1H‐benzimidazol and sprayed with *Bgt* spores (isolate E09) using an air compressor and nozzle. After 3 d, the leaves were bleached with trichloroacetic acid (1.5 g l^−1^) in ethanol : chloroform (4 : 1 v/v), stained with aniline blue (1 g l^−1^), and observed under a light microscope for the formation of elongated secondary hyphae (Li *et al*., 2005). A segment of the same leaf was stored at −80°C for mRNA extraction and subsequent qRT‐PCR.

### Single‐cell transient gene expression assay

The single‐cell transient gene expression assay was conducted according to Shen *et al*. (2007). The coding sequence (CDS) region of *AeGlu* was cloned into a vector under the control of the maize poly‐ubiquitin promoter. The pUbi−AeGlu constructs were mixed with a β‐glucuronidase (GUS) reporter vector at a 1 : 1 molar ratio and used to coat DNA microcarriers, which were delivered into wheat leaf epidermal cells by particle bombardment (Bio‐Rad, Model PDS‐1000/He). The leaves were treated with *Bgt* conidia spores at 4 h after bombardment. GUS activity was detected in the leaves 2 d later by GUS staining, followed by incubation in destaining solution. Fungal epiphytic structure was observed after staining the leaves with Coomassie blue, and the fungal haustorium index was scored.

## Results

### *Bgt* Infection represses the expression of *AGO4* genes

**Figure 1:**
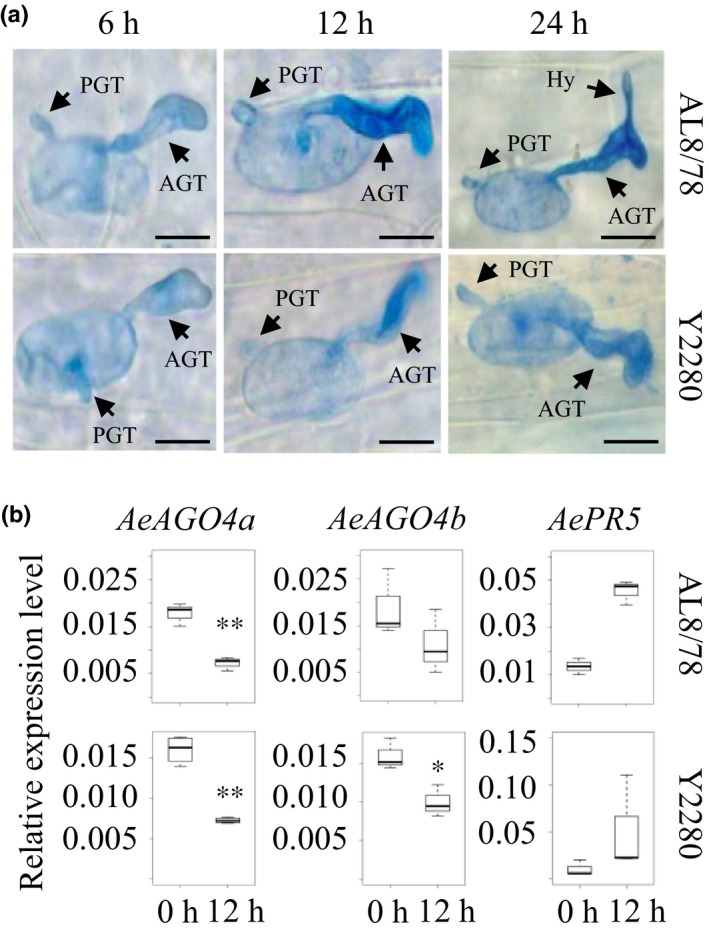
Morphological development of *Blumeria graminis* f. sp. *tritici* (*Bgt*) during compatible and incompatible interactions with *Aegilops tauschii* and expression patterns of *AeAGO4* genes at 12 h after inoculation (hai). (a) Microscopy observation of the morphology of *Bgt* during growth on the leaves of the *Bgt‐*susceptible line AL8/78 and *Bgt‐*resistant line Y2280 at 6, 12 and 24 hai. PGT, primary germ tube; AGT, appressorium germ tube; Hy, secondary hyphae. Bars, 20 μm. (b) Expression of *AeAGO4a* and *AeAGO4b* in *A. tauschii* following *Bgt* inoculation. Leaves of 2‐wk‐old AL8/78 and Y2280 plants were inoculated with *Bgt* for 12 h, and mRNA levels of *AeAGO4a* and *AeAGO4b* were measured. Pathogenesis‐related gene *AePR5* was used as an indicator of successful inoculation. Student's *t*‐test: *, *P *<* *0.05; **, *P *<* *0.01. The top bar represents the maximum of all the data and the bottom bar represents the minimum of all the data.

To determine the optimal time point for an effective interaction between *A. tauschii* and *Bgt*, we monitored the growth conditions of *Bgt* on two *A. tauschii* accessions, AL8/78 and Y2280, which are susceptible and resistant, respectively, to *Bgt* race no. 15 from the Beijing area. Typical white, powdery pustules and symptoms were observed on the leaves of AL8/78, but not Y2280, at 7 d after inoculation (dai) (Fig. S1). Secondary hyphae developed at *c*. 24 hai during the compatible interaction with AL8/78, but not with Y2280 (Fig. 1a), indicating successful infection of *Bgt* in AL8/78. As 12 hai was the earliest time point when *Bgt* displayed growth differences between compatible and incompatible interactions with *A. tauschii*, we selected leaf tissues at 12 hai for transcriptome analysis to identify host genes with altered expression levels in response to *Bgt* inoculation.

In rice, two AGO4 homologs, AGO4a and AGO4b, are essential for 24‐nt siRNA sorting via the RdDM pathway (Wu *et al*., 2010). We identified AGO4a and AGO4b homologs in *A. tauschii* as well (Fig. S2; Table S2). qRT‐PCR analysis showed that *AGO4a* was significantly downregulated at 12 hai with *Bgt* in the compatible interaction with AL8/78 and incompatible interaction with Y2280 (Student's *t*‐test, *P *<* *0.01), while AGO4b showed significant differences only during incompatible interactions (Fig. 1b), indicating that *AGO4a* expression is independent of host genetic background and *Bgt* compatibility. Expression analysis of the late defense‐marker gene *Pathogenesis‐related gene 5* (*PR5*) showed that this gene was significantly induced in both AL8/78 and Y2280 at 12 hai (Fig. 1b). Similar responses for *PR10* genes were found in common wheat, as all three homoeologs of *AGO4a* were downregulated upon *Bgt* inoculation (Fig. S3). These observations suggest that AGO4a‐related mechanisms for *Bgt* defense responses may be conserved in *A. tauschii* and common wheat.

### Reduced levels of AGO4a‐associated 24‐nt siRNAs in response to *Bgt* infection

The main function of AGO4 proteins is to load 24‐nt siRNAs in order to guide the RNA‐induced silencing complex (RISC) complex for sequence‐specific DNA methylation (Matzke & Mosher, 2014; Deleris *et al*., 2016). We thus examined whether AGO4‐associated 24‐nt siRNAs were also affected upon *Bgt* infection in *A. tauschii*.

**Figure 2:**
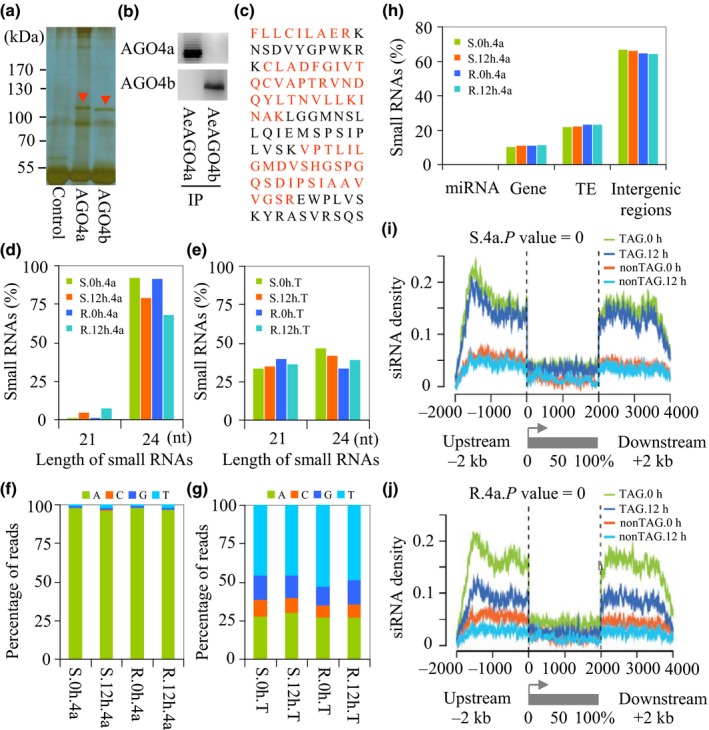
Characterization of small RNAs associated with AGO4 proteins during *Aegilops tauschii* and *Blumeria graminis* f. sp. *tritici* (*Bgt*) interactions. (a) AGO4 clade proteins AGO4a and AGO4b were immuno‐purified from total leaf proteins using peptide‐specific antibodies and separated on a 10% SDS‐PAGE gel. Pre‐immune antisera were used as a control. The proteins were visualized by silver staining. The two red arrows indicate AGO4a and AGO4b protein bands (predicted based on their molecular sizes), which were absent from the control. The positions of protein size markers, electrophoresed in parallel, are shown to the left of the gel. (b) Immunoblot analysis of AGO4a‐ and AGO4b‐specific antibodies in AL8/78. Samples were collected from the leaves of 2‐wk‐old AL8/78 plants; AGO4a and AGO4b were detected using the corresponding antibodies. Similar results were obtained from two replicates. (c) Characterization of purified AeAGO4a by mass spectrometry, with the detected peptides marked in red. (d, e) Changes in AGO4a‐associated small RNAs pulled down by (d) AGO4a and (e) total sRNAs from proteins isolated from AL8/78 (S) and Y2280 (R) leaves at 12 h after inoculation (hai) with *Bgt*. (f, g) Frequency of the first nucleotide of 24‐nt small RNAs pulled down by (f) AGO4a and (g) in the total 24‐nt small RNAs from AL8/78 (S) and Y2280 (R) leaves at 12 hai with *Bgt*. (h) Distribution of small RNAs pulled down by AGO4a‐specific antibody from different components of the *A. tauschii* genome. (i, j) Small RNA densities at TE‐associated genes (TAG) and non‐TAGs isolated by AGO4a‐specific antibody from (i) AL8/78 and (j) Y2280 leaves at 12 hai with *Bgt*. *P*‐values were derived from a Wilcoxon paired ranks sum test. Sliding windows for small RNAs were 100 bp with 10‐bp steps.

We generated two antibodies against the N‐terminal peptides of *A. tauschii* AGO4 proteins (AeAGO4a and AeAGO4b). Enriched bands were clearly observed on a polyacrylamide gel (Fig. 2a). The specificity of these antibodies was confirmed by immunoblot analysis of leaf proteins from plants with and without a 12‐h *Bgt* treatment (Figs 2b, S4). Mass spectrometry of gel‐purified proteins revealed peptide sequences identical to the predicted AGO4a sequence (Figs 2c, S5; Table S3), but only a few peptides were identified for AGO4b (Fig. S5; Table S3), indicating that the AGO4a antibody was more efficient than the AGO4b antibody.

For sRNA analysis, total proteins were isolated from equal amounts of treated (12 hai with *Bgt*) and mock‐treated leaf tissues from 2‐wk‐old plants. The sRNAs were then cross‐linked with the associated proteins and pulled down by immuno‐precipitation using the AeAGO4a and AeAGO4b antibodies. Illumina sequencing resulted in an average of *c*. 10 million total reads per sample. Reads 19‐ to 28‐nt long were separated, comprising 7–18 million reads per sample. Approximately 78–94% of these reads mapped perfectly to the *A. tauschii* genome and were subsequently analyzed (Table S4).

The 24‐nt sRNAs were the most abundant sRNAs pulled down with AGO4a, representing 68–92% of the total (Fig. 2d). By contrast, in the absence of a pull‐down step, the percentages of 21‐ and 24‐nt sRNAs were similar, representing 33–39% and 34–46% of total sRNAs, respectively (Fig. 2e). Interestingly, *Bgt* infection led to a 13% reduction in 24‐nt siRNA levels in AL8/78 compared with the mock (92% vs 79%), with a greater reduction detected in Y2280 (24%, or 92% vs 68%) (Fig. 2d). Further analysis showed that AGO4a predominantly (> 96%) bound to sRNAs whose first base was adenine (A) (Fig. 2f), whereas no such specificity was found among AGO4b‐associated siRNAs (Fig. S6) or among total sRNAs (Fig. 2g). These results indicate that AeAGO4a is likely to be the ortholog of Arabidopsis AGO4 that carries a specific class of 24‐nt sRNAs, as in other plant species (Mi *et al*., 2008; Wu *et al*., 2010). Moreover, *AGO4a* responded to *Bgt* infection more strongly than did *AGO4b* (Fig. 1b). These observations support the notion that AGO4a is the major AGO4 protein involved in DNA methylation. Thus, AGO4a was the focus of subsequent analysis.

Most AGO4a‐associated 24‐nt sRNAs (65.8%) were located in intergenic regions (Fig. 2h): 22.8% of 24‐nt siRNAs were found in regions identified as TEs, while 11.1% were located in genic regions. The regulation of TEs by DNA methylation can affect the expression of nearby genes (Deleris *et al*., 2016). Using a method described by Li *et al*. (2014), we found that up to 70% (10 811) of gene models co‐located with TEs within their neighboring regions (including 2‐kb 5′ and 3′ regions) in the *A. tauschii* genome (Fig. S7). These genes were designated as TE‐associated genes, or TAGs. Of the four major groups of TEs identified, more were located in the 5′ upstream and 3′ downstream regions than in coding regions, and > 30% of these were miniature inverted‐repeat elements (MITES) (Fig. S8). We plotted the density of 24‐nt siRNAs along these TAGs. As shown in Fig. 2(i), significant differences in 24‐nt siRNA levels (Wilcoxon paired ranks sum test, *P *=* *0) were found between AL8/78 leaf tissues collected at 0 hai and 12 hai with *Bgt*. By contrast, there were no significant differences in 24‐nt siRNA levels at non‐TAG gene models (Fig. 2i; Table S5). The difference appeared to be more significant in Y2280 (Wilcoxon paired ranks sum test, *P *=* *0), which is resistant to the *Bgt* race used, than in the other accession (Fig. 2j; Table S5). These results indicate that *Bgt* inoculation indeed reduces AGO4a‐associated 24 nt siRNA levels in *A. tauschii*.

### The regulation of host DNA methylation in response to *Bgt* inoculation

**Figure 3:**
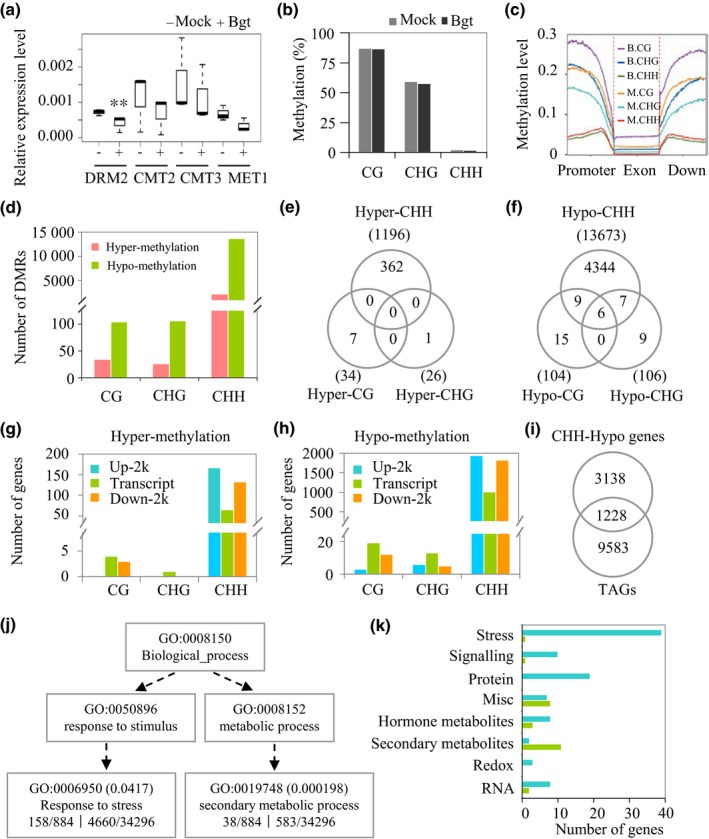
The effect of *Blumeria graminis* f. sp. *tritici* (*Bgt*) infection on DNA methylation in *Aegilops tauschii* during the compatible interaction. (a) Expression patterns of DNA methylase genes at 12 h after inoculation (hai) with: *Bgt*, RNA sample from leaves at 12 hai with *Bgt*; Mock, RNA sample harvested 12 h after water treatment. Student's *t*‐test: **, *P *<* *0.01. The top bar represents the maximum of all the data and the bottom bar represents the minimum of all the data. (b) Percentages of cytosine methylation in the three contexts. (c) The density of methylated cytosines at the genic regions. Sliding windows are 100 bp with 10‐bp steps. (d) Total number of differentially methylated regions (DMRs) between *A. tauschii* genomes treated with *Bgt* (Bgt) and water (mock). Differences between the two conditions for CpG, CHG, and CHH were set to 0.4, 0.2, and 0.1, respectively (see the Materials and Methods section). (e, f) Venn diagrams showing the number of genes overlapping with (e) hypermethylated and (f) hypomethylated DMRs for all three methylation contexts. The total number of DMRs of each context is shown in parentheses. (g, h) Number of genes with (g) hypermethylated and (h) hypomethylated DMRs at genic regions. Transcript, open reading frame (ORF); up‐2k, 2 kb upstream of the ORF; down‐2k, 2 kb downstream of the ORF. (i) Venn diagram showing the overlap of genes with CHH‐hypomethylated DMRs and transposable element (TE)‐associated genes (TAGs). (j) Gene Ontology (GO) enrichment analysis of 1228 genes associated with TEs and CHH‐hypomethylated DMRs. (k) MapMan classification of genes from GO:0006950 (blue bars) and GO:0019748 (green bars) in (j).

DNA methylation is mediated by 24‐nt siRNA in the RdDM pathway, which often induces the silencing of nearby genes. We found that the levels of AGO4a‐associated 24‐nt siRNAs were reduced in *A. tauschii* leaves inoculated with *Bgt*. To determine whether genes directly involved in DNA methylation are affected by *Bgt* inoculation, we examined the expression levels of DNA methylase genes during the *A. tauschii*–*Bgt* interaction. As shown in Figs 3(a) and S9, the expression of the DNA methylase homolog *DRM2* was significantly (Student's *t*‐test, *P *<* *0.01) reduced by *Bgt* inoculation in both compatible and incompatible interactions. The downregulation of other DNA methylase gene homologs, such as *CMT2, CMT3*, and *MET1* was not statistically significant. Interestingly, a similar pattern was observed in common wheat (Fig. S10), indicating that the response of DNA methylase gene homologs to *Bgt* infection is conserved between these two species. These observations encouraged us to examine the *A. tauschii* methylomes during these interactions.

We developed and sequenced two bisulfite‐treated DNA libraries to compare the methylation levels of mock (M12) vs 12 hai (B12) samples using AL8/78, because it is compatible with the *Bgt* race used in this study, and its genome has been sequenced (Jia *et al*., 2013; Zhao *et al*., 2017). In total, 1222 million and 824 million reads were produced for M12 and B12, respectively (Table S6). Among these, more than 629 million and 428 million reads were uniquely aligned to the *A. tauschii* reference genome for M12 and B12, respectively. The bisulfite conversion efficiency was high (>99%; Table S6). In both M12 and B12, the most highly methylated sites were CG sites (87.1% and 86.4%, respectively), while 59.4% and 57.6% CHG sites, respectively, were methylated. Among the CHH sites, 1.9% were methylated in M12 and 1.6% were methylated in B12 (Fig. 3b; Table S6). As DNA methylation‐regulated gene expression is primarily achieved by modifying the methylation status of sequences close to their coding regions, we examined the density of methylated cytosines along genic regions. The average methylation levels at genic regions were much lower than those detected at the genome‐wide level. Moreover, coding regions had much lower methylation levels than 5′ upstream and 3′ downstream regions (Fig. 3c).

We then determined the cytosine methylation levels in each 100‐bp window along the genome using the method of Stroud *et al*. (2013). Genomic regions with a methylation difference of 0.4, 0.2, and 0.1 for CG, CHG, and CHH, respectively, were considered DMRs, with an adjusted *P*‐value (FDR) of < 0.01 (Fisher's exact test). DMRs located within 100 bp of each other were merged (Stroud *et al*., 2013). Under these conditions, only a few DMRs were found for CG (138) and CHG (132) methylation. By contrast, there were significantly more CHH DMRs (14 869), despite their lower methylation level compared with CG and CHG DMRs (Fig. 3d; Tables S7, S8). These data demonstrate that CHH sites are the main loci for the regulation of DNA methylation during *A. tauschii*–*Bgt* interactions.

We then screened for genes that were associated with DMRs at their genic regions. Many more genes (4366) were associated with CHH‐hypomethylated DMRs vs CHH‐hypermethylated DMRs (362; Fig. 3e,f). Among genes with three DMR contexts (CG, CHG, CHH), only six genes were common among hypomethylated DMRs, while none was common among hypermethylated DMRs. We then identified DMR locations relative to gene‐coding regions. As shown in Fig. 3g and h, more CHH DMRs were located in upstream and downstream regions of genes than in coding regions for both hypermethylated and hypomethylated DMRs. This observation is consistent with findings in rice and soybean (*Glycine max*) (Rambani *et al*., 2015; Tan *et al*., 2016). We then overlapped the CHH‐hypomethylated genes and TAGs and found that 28% (1228) of genes with CHH DMRs were associated with TE(s) (Fig. 3i; Table S9). GO enrichment analysis showed that the overlapping genes (1228) were enriched for ‘secondary metabolic processes’ and ‘stimulus stress’ functions (Fig. 3j). Further examination of the genes by MapMan analysis showed that they fell into multiple functional categories, including stress, signaling, and cell wall pathways that are potentially involved in pathogen defense (Fig. 3k; Table S10).

To validate the effects of CHH methylation on gene expression, we performed qRT‐PCR analysis of a set of genes selected from the MapMan stress bin. We selected 15 genes with various TEs and CHH‐hypomethylated DMRs relative to coding regions (Fig. S11). Among the six TAGs with CHH‐hypomethylated DMRs, three (AEGTA32510, 11287, and 12875) were clearly upregulated, one (AEGTA31633) was expressed at low levels during the first 24 h and upregulated at 48 hai, and two (AEGTA08756, 04989) were downregulated and maintained low expression levels at all time points. Of the nine remaining genes, seven were upregulated at at least one time point, while two (AEGTA02546, 09103) were downregulated at every time point. These results demonstrate that, despite the complex expression patterns of genes associated with DMRs, the expression patterns of at least some of the genes at certain stages were coordinated with their CHH methylation status during the response of *A. tauschii* to *Bgt* infection. A correlation test revealed a significant negative correlation between the extent of methylation and the expression levels of these genes (Pearson correlation coefficient *r* = −0.68, Student's *t*‐test, *P *<* *0.01) (Fig. S12).

### Downregulation of *DRM2* enhances *Bgt* defense in *A. tauschii* during compatible interactions

**Figure 4:**
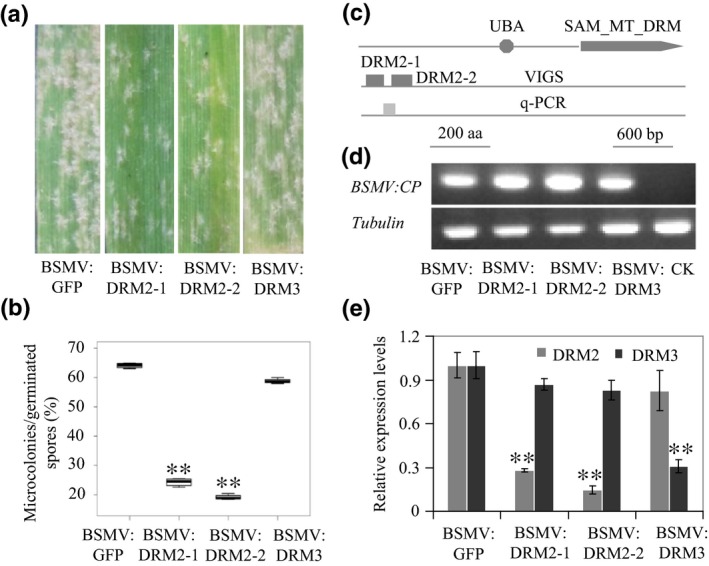
Knockdown of *DRM2* by virus‐induced gene silencing (VIGS) enhances *Blumeria graminis* f. sp. *tritici* (*Bgt*) defense in AL8/78. (a) *Bgt* inoculation and microcolony growth on AL8/78 leaves of two *DRM2* VIGS lines (BSMV:DRM2‐1 and BSMV:DRM2‐2). Plants were grown for 7 d after inoculation. Plants infected by BSMV:GFP and BSMV:DRM3 were used as nontarget controls. (b) Percentages of successfully colonized *Bgt* microcolonies out of all analyzed spores on the *GFP* and *DRM3* control and *DRM2* VIGS plants. Leaf segments 3–4 cm long were collected from the fourth leaves of control and VIGS plants. The top bar represents the maximum of all the data and the bottom bar represents the minimum of all the data. (c) Gene structure of *DRM2*, as predicted by ScanProsite (http://prosite.expasy.org/scanprosite/). UBA, ubiquitin‐associated domain. SAM_MT_DRM, SAM‐dependent methyltransferase DRM‐type domain: Boxes labeled DRM2‐1 and DRM2‐2, DNA fragments used for VIGS construct development; gray box, regions used for qPCR. (d) RT‐PCR analysis of relative transcript levels of the BSMV *COAT PROTEIN* (*CP*) gene in the fourth leaves of BSMV:DRM2 and the control (CK, GKP buffer). Amplification of the wheat tubulin gene was used as an internal control. (e) qRT‐PCR assay showing downregulation of *DRM2* expression in AL8/78 control and VIGS plants. Leaves from at least 15 plants were tested for each vector. Error bars indicate ± SD of three independent experiments. Student's *t*‐test: **, *P *<* *0.01.

**Figure 5:**
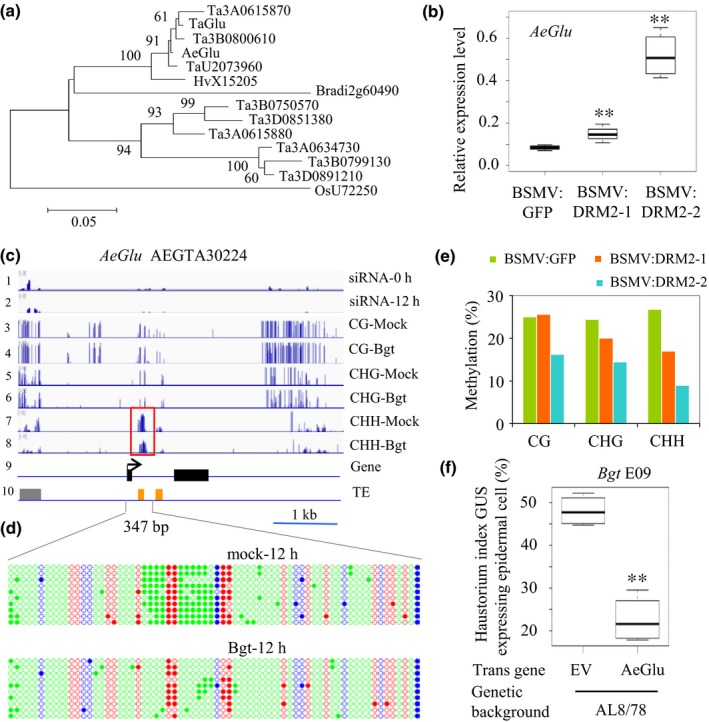
Characterization of the transposable element (TE)‐associated PR‐related gene *AeGlu* in response to *Blumeria graminis* f. sp. *tritici* (*Bgt*) infection. (a) Phylogenetic analysis of endo‐1,3‐β‐d‐glucosidase (Glu) family proteins, showing that *AeGlu* (genome annotation, AEGTA30224) is a potential ortholog of a *PR2* gene. The midpoint‐rooted phylogenetic tree was constructed using Mega (v.5.0) and the Neighbor‐Joining method. The equal input substitution model was used with bootstrap values generated from 1000 trials. The evolutionary distances were computed in number of amino acid substitutions per site, as shown by the scale below the tree. (b) Relative transcript levels of *AeGlu* in control and *DRM2* virus‐induced gene silencing (VIGS) plants. (c) Gene structure of *AeGlu* (line 9, black boxes represent exons) and its associated TEs (line 10; yellow boxes, TcMar‐Stowaway; gray box, LINE/L1). Above the gene structure are the distribution and density of 24‐nt small RNAs (lines 1 and 2), CG methylation (lines 3 and 4), CHG methylation (lines 5 and 6), and CHH methylation (lines 7 and 8) of AL8/78 leaves treated with *Bgt* (Bgt) for 12 h vs mock treatment. (d) Confirmation of differential CHH methylation at the MITE region by PCR amplification and sequencing of bisulfite‐treated DNA from 12 h after inoculation (hai) AL8/78 leaves and the mock control. Colors represent different methylation contexts: red, CG; blue, CHG; green, CHH. Cytosines are indicated by open circles when nonmethylated and closed circles when methylated. (e) Decreased DNA methylation at the corresponding region of *AeGlu* in (d). BSMV:DRM2‐1 and BSMV:DRM2‐2 are VIGS lines and BSMV:GFP is the control. (f) *AeGlu* overexpression enhances the resistance of AL8/78 against *Bgt*. Empty vector (EV) was used as a control. Student's *t*‐test: **, *P *<* *0.01. (b, f) The top bar represents the maximum of all the data and the bottom bar represents the minimum of all the data.

To further demonstrate that reduced DNA methylation plays a role in the *Bgt* defense response in *A. tauschii*, we downregulated the expression of a homolog of the major CHH methylase gene, *DRM2* (Fig. S13), in AL8/78 using VIGS. As shown in Fig. 4, inoculated *DRM2* VIGS plants had fewer microcolonies than the control (Fig. 4a); these differences were significant among total germinated spores in A8/L78 (Fig. 4b), indicating enhanced resistance to *Bgt* infection. Figs 4(c) and S14 show the locations of the fragments used for VIGS construct development. BSMV infection was successful, as indicated by the expression of the BSMV coat protein gene (Fig. 4d). qRT‐PCR analysis showed that endogenous *DRM2* was indeed significantly downregulated in BSMV:DRM2 VIGS plants, while the expression of the *DRM2* homolog *DRM3* was not significantly affected (Fig. 4e). In addition, several biotic response‐related genes were significantly upregulated in these plants (Fig. S15), including the PR‐related β‐1, 3‐glucanase gene *AeGlu* (AEGTA30224) (Figs 5a,b, S16, S17). A homolog of *AeGlu* was shown to act alone or in combination with chitinase during the fungal defense response in common wheat and other species (Liu *et al*., 2010). The upregulation of these genes may contribute to *Bgt* defense in BSMV:DRM2 VIGS plants.

### Reduced CHH methylation at *AeGlu* may be responsible for enhanced resistance to *Bgt*

To provide more evidence that changes in CHH methylation affect *Bgt* defense responses, we examined the genomic structure of the *PR2*‐like gene *AeGlu* in AL8/78. We detected one LINE/L1‐like TE in the promoter region and two stowaway‐like MITES in the first intron (Fig. 5c). The MITE region corresponded to a CHH‐hypomethylated DMR where CG and CHG methylation was not altered. Changes in siRNA density were found at the 5′ region of the gene corresponding to the location of the LINE/L1‐like TE upon *Bgt* infection. In rice, MITEs are the most abundant short TEs in genic regions (Bureau & Wessler, 1994; Zemach *et al*., 2010), and CHH methylation patterns parallel the distribution of MITEs (Tan *et al*., 2016). Our findings are consistent with these observations, suggesting that the regulation of *AeGlu* expression may indeed involve DNA methylation at associated MITE loci. We further confirmed the Methyl‐Seq results by PCR amplification of the MITE region from bisulfate‐treated DNA and by sequencing. As shown in Fig. 5(d), CHH methylation was clearly reduced at this region, while changes in CG and CHG methylation were not significant.

We also observed changes in DNA methylation at the *AeGlu* locus in *DRM2* VIGS plants. CHH methylation was significantly reduced in these plants, as determined by PCR amplification of the MITE region from bisulfate‐treated VIGS plant DNA and by sequencing. As shown in Fig. 5(e), the change in CHH methylation level in the MITE region in the *DRM2* VIGS plant DRM2‐1 was *c*. 10% (0.1), and that in DRM2‐2 was >20% (0.2) (Figs 5(e), S18). Notably, CG and CHG methylation levels were also reduced in the region (by *c*. 10%; 0.1). As the overall DNA methylation levels at CG and CHG were high across the genome, the changes in the levels of these two types of methylation were not considered significant. Together, these results suggest that the reduced CHH methylation at *AeGlu* may contribute to the upregulation of this *PR2*‐like gene. Indeed, we measured the expression level of *AeGlu* in *Bgt*‐challenged DRM2‐VIGS plants and found that it was strongly upregulated in both green fluorescent protein (GFP) and DRM2 VIGS plants, especially the latter (Fig. S19).

We further explored the role of *AeGlu* in *Bgt* defense by performing single‐cell transient gene expression assays. As shown in Fig. 5f, co‐expression of *AeGlu* with the GUS reporter gene in AL8/78 leaf epidermal cells resulted in a marked reduction in the haustorium index (HI%; 22%) upon inoculation with virulent *Bgt* conidiospores. This result is in clear contrast with that of the empty vector control, whose HI% was much higher (48%; Fig. 5f). Thus, the regulation of PR2‐like genes such as *AeGlu* by DNA methylation at least partially contributes to enhanced fungal defense in *A. tauschii*.

## Discussion

### The role of 24‐nt siRNAs in responses to pathogen infection

Increasing evidence indicates that epigenetic regulation is another important gene regulatory mechanism for plant immunity (Ding & Wang, 2015). During this process, differential expression of endogenous sRNAs accompanies defense responses to pathogen attack (Katiyar‐Agarwal & Jin, 2010). Studies of sRNA biogenesis and sRNA functioning genes have shown that impairing these genes reduces the defense against certain pathogens (Weiberg & Jin, 2015). We show here the *A. tauschii* AGO4a antibody specifically pulled down 24‐nt siRNAs that began with the base adenine, a typical characteristic of AGO4a proteins, as observed in Arabidopsis (Mi *et al*., 2008) and rice (Wu *et al*., 2010). Upon infection with the biotrophic fungal pathogen *Bgt*,* AGO4a* was significantly downregulated, a response similar to that in Arabidopsis plants elicited by the bacterial PAMP flg22 (Yu *et al*., 2013). As AGO4a and 24‐nt siRNAs are major components of the RdDM pathway, which methylates TEs near genes, the downregulation of these components suggests the presence of an active defense mechanism in *A. tauschii* that coordinately downregulates DNA methylation, hence upregulating defense gene expression. In Arabidopsis, DNA demethylation functions may prime transcriptional activation of defense genes with TEs/repeats (Yu *et al*., 2013). Nevertheless, in light of the current findings, the possibility that the pathogen manipulates the expression of these genes and sRNA biosynthesis cannot be completely ruled out. Such mechanisms indeed exist during plant–pathogen interactions, such as the secretion of ‘virulent’ sRNA effectors by the pathogen, which function as suppressors of host immunity to achieve infection; this is an example of an advanced virulence mechanism involving naturally occurring cross‐kingdom RNAi (Weiberg *et al*., 2013). Whether fungal sRNA downregulates *AGO4a* and reduces total siRNA/CHH methylation levels during *Bgt*–wheat interaction requires further study.

The differential expression levels of 24‐nt siRNAs in compatible vs incompatible interactions with *Bgt* reinforce the idea that sRNAs play critical roles in the basal defense responses by guiding DNA methylation. Upon *Bgt* infection, the incompatible cultivar appeared to accumulate less siRNA than the compatible cultivar (Fig. 2d). There are several possible explanations for this observation. On the one hand, genes involved in 24‐nt siRNA production, such as *POL IV*,* RDR2*, and *DCL3a*, were more strongly downregulated in the incompatible interaction with *Bgt* (Y2280) compared with the compatible interaction (Fig. S20). On the other hand, genes encoding siRNA loading proteins such as AGO4a and AGO4b were also more severely downregulated in Y2280 (incompatible; Fig. 1b), pulling down fewer siRNAs than those from AL8/78. Notably, in Arabidopsis, DNA methylation may also be mediated by salicylic acid‐induced TAG‐derived 21‐nt sRNA (Dowen *et al*., 2012), a mechanism clearly different from that found in the current study. Our conclusions are more similar to those of Yu *et al*. (2013) that DNA demethylation plays a role in activating TE‐associated defense genes. As several key genes involved in the RdDM were affected by pathogen infection, it is crucial to determine the extent of the role of the RdDM pathway in temperate grass immunity.

### CHH methylation as targets of regulation

Compared with CG and CHG sites, CHH sites had extremely low levels of genome‐wide methylation. Nonetheless, most DMRs (> 90%) were derived from CHH sites, and their levels were reduced (by >10%) in response to *Bgt* infection. This result is in line with previous studies showing that, among the three cytosine methylation contexts, the regulation of CHH methylation occurs most frequently at genic regions (Li *et al*., 2015; Rambani *et al*., 2015; Tan *et al*., 2016). CHH methylation requires the RdDM pathway for *de novo* methylation (Matzke & Mosher, 2014), and its maintenance also requires the RdDM pathway, especially at sites near genes marked by H3K9me2 (Law *et al*., 2013). Therefore, CHH methylation is mediated by the RdDM pathway, and may represent the first or most sensitive target of regulation during the response to pathogen infection. The overall lower methylation levels at CHH sites may provide targets for precise gene regulation under stress conditions.

Detailed observations in maize, another species with a high repetitive sequence content, show that, unlike genes that have a high level of CG and CHG methylation, genes that are close to CHH islands, which have a high proportion of CHH methylation, are more likely to be coordinated by the methylation status of immediately adjacent TEs (Gent *et al*., 2013). However, the role of CHH regulation may be limited, as the expression of only a portion of genes appear to be coordinated with their DNA methylation status. Other regulatory modes might also exist, such as regulation by transcription factors.

### The regulation of DNA methylation in plants in response to pathogen infection

Changes in DNA methylation and demethylation enhance tolerance to the bacterium *Pst* DC3000 in Arabidopsis (Yu *et al*., 2013). Similarly, in rice, the application of 5‐azadeoxycytidine, a DNA demethylating agent, enhances tolerance to *X. oryzae* (Akimoto *et al*., 2007). Treatment with PAMPs, such as flg22, results in the suppression of TGS leading to de‐repression of RdDM targets. Mutations in other RdDM components, such as Pol V, also cause changes in pathogen responses, pointing to their possible role in plant immunity (López *et al*., 2011; Matzke & Mosher, 2014). In *A. tauschii*, a significant number of CHH DMRs are located at genic regions, especially 5′ and 3′ genomic regions. As these areas might represent heterochromatic and euchromatic boundaries, the changes in CHH methylation levels probably have both structural and functional roles, as shown in maize (Li *et al*., 2015).

In this study, we obtained further evidence that DNA methylation dynamics contribute to fungal defense using a diploid progenitor of common wheat as a model. We obtained results comparable with those from other plant species. The disruption of DNA methylation via VIGS‐mediated downregulation of *DRM2* activated the expression of *PR2* genes. This study is the first of its kind using temperate grasses interacting with a biotrophic fungal pathogen.

It should be noted that although DNA methylation appears to negatively regulate bacterial and biotrophic fungal resistance, it might positively regulate resistance to other types of pathogens, such as necrotrophic fungi. Indeed, RdDM‐defective mutants are more susceptible than the wildtype to *Botrytis cinerea* and *Plectosphaerella cucumerina* (López *et al*., 2011). This observation points to diverse, complex mechanisms underlying the role of DNA methylation in regulating plant immunity. Nonetheless, our findings indicate that DNA methylation functions in plant defense responses to obligate biotrophic fungi. At least some, if not all, defense‐related genes are upregulated upon *Bgt* infection, which causes DNA hypomethylation, especially CHH hypomethylation, via TEs.

### Application of these mechanisms to common wheat

Common wheat is a hexaploid plant that originated from a hybridization event that occurred *c*. 8000 years ago (Tanno & Willcox, 2006). In addition to their genetic compatibility (usually achieved by artificial crossing), the DNA sequences of the A, B, and D subgenomes are highly similar, with an average of up to 94% nucleotide sequence identity. Therefore, functional analysis of genes in *A. tauschii* can shed light on similar processes in common wheat. Indeed, the transfer of functional loci via distant hybridization has been widely used in wheat for genes such as *Sr35*. Transfer of *Sr35* imparted resistance to the newly emerged Ug99 race of *Puccinia graminis* f. sp. *tritici* (*Pgt*), which causes severe stem rust and yield losses. *Sr35* was first cloned from *T. monococcum*, another diploid wheat relative with an AA genome, and then transferred into hexaploid wheat by distant hybridization (Saintenac *et al*., 2013). Similarly, the information obtained here by studying DNA methylation patterns in *A. tauschii* during interactions with *Bgt* should be applicable to common wheat. Indeed, the *Bgt* race used in this study can cross‐infect *A. tauschii* and common wheat. Moreover, *AGO4a* and *DRM2*, two key genes in the RdDM pathway, were downregulated upon *Bgt* infection in both species. These common phenotypes and molecular reactions point to conserved defense mechanisms in common wheat and its diploid progenitor. Therefore, efforts to understand the epigenetic mechanism of *Bgt* defense responses in *A. tauschii* should give rise to novel strategies to improve disease resistance in common wheat.

## Author contributions

XL, AL and LM planned and designed the research; SG, XK, GS and MJ performed the experiments; JG, FW, AL and LM analyzed the data; ZQ and LW provided technical assistance; and SG, AL and LM wrote the article, with contributions from all of the authors. SG and XK contributed equally to this work.

## Supporting information

Please note: Wiley Blackwell are not responsible for the content or functionality of any Supporting Information supplied by the authors. Any queries (other than missing material) should be directed to the *New Phytologist* Central Office.

**Fig. S1** Response of *Aegilops tauschii* to *Bgt* inoculation.
**Fig. S2** Phylogenetic relationships of plant AGO4a and AGO4b proteins.
**Fig. S3** Expression of *AGO4a* homoeologs and *TaPR10* in bread wheat following *Bgt* inoculation.
**Fig. S4** Immunoblot analysis of AGO4a‐ and AGO4b‐specific antibodies in AL8/78.
**Fig. S5** Mass spectrometry analyses of purified *A. tauschii* AGO4 proteins.
**Fig. S6** Characterization of 24‐nt sRNAs pulled down by AGO4b in *A. tauschii*.
**Fig. S7** Proportions of TAGs and locations of TEs in *A. tauschii* gene models.
**Fig. S8** Distribution of TEs relative to genic regions of TE‐associated genes in the genome of *A. tauschii.*
**Fig. S9** DNA methylase gene expression patterns in the *A. tauschii*–*Bgt* incompatible interaction.
**Fig. S10** Expression patterns of DNA methylase genes upon *Bgt* inoculation in bread wheat.
**Fig. S11** Expression patterns of genes with CHH‐hypomethylated differentially methylated regions (DMRs).
**Fig. S12** Relationship between CHH methylation extent and the expression patterns of various genes after *Bgt* inoculation in AL8/78.
**Fig. S13** Phylogenetic relationships of plant DRM2‐related proteins.
**Fig. S14** Gene structure of *DRM3*.
**Fig. S15** Characterization of two DMR genes in response to *Bgt* infection.
**Fig. S16** Expression patterns of *AeGlu* upon *Bgt* inoculation.
**Fig. S17** Multiple sequence alignment of endo‐1,3‐beta‐d‐glucosidase genes from wheat‐related species.
**Fig. S18** Detection of methylation status at the *DRM2* gene regions (Fig. 5d) in VIGS plants at 12 hai with *Bgt*.
**Fig. S19** Relative transcript levels of *AeGlu* in mock‐treated (GFP) and DRM2 VIGS AL8/78 plants following *Bgt* inoculation.
**Fig. S20** Expression of *AePOL IV*,* AeRDR2*, and *AeDCL3a* in *A. tauschii* following *Bgt* inoculation.
**Table S1** List of primers used in this study.
**Table S2** Major components of the RNA‐directed DNA methylation (RdDM) pathway in rice and their homologs in *A. tauschii* and wheat.
**Table S3** AeAGO4a and AeAGO4b peptides identified by mass spectrometry.
**Table S4** Statistics of raw small RNA reads and processing results.
**Table S5** Differential expression of 24‐nt siRNAs pulled down by AGO4a antibody in AL8/78 and Y2280.
**Table S6** Summary of cytosine methylation status in mock‐ and *Bgt*‐inoculated samples.
**Table S7** List of DMRs in three methylation contexts.
**Table S8** List of unique methylated genes.
**Table S9** List of TAGs bearing CHH‐hypomethylated DMRs.
**Table S10** Genes potentially involved in pathogen defense revealed by the MapMan program.
