# Supplementary material for: DNA methylation dynamics during the interaction of wheat progenitor Aegilops tauschii with the obligate biotrophic fungus Blumeria graminis f. sp. tritici
Source: New Phytol. 2018 Sep 6;221(2):1023–35. doi: 10.1111/nph.15432 (PMC6586159; doi:10.1111/nph.15432)
Supplement: Supplementary file 1 — Fig. S1 Response of Aegilops tauschii to Bgt inoculation. Fig. S2 Phylogenetic relationships of plant AGO4a and AGO4b proteins. Fig. S3 Expression of AGO4a homoeologs and TaPR10 in bread wheat following Bgt inoculation. Fig. S4 Immunoblot analysis of AGO4a‐ and AGO4b‐specific antibodies in AL8/78. Fig. S5 Mass spectrometry analyses of purified A. tauschii AGO4 proteins. Fig. S6 Characterization of 24‐nt sRNAs pulled down by AGO4b in A. tauschii. Fig. S7 Proportions of TAGs and locations of TEs in A. tauschii gene models. Fig. S8 Distribution of TEs relative to genic regions of TE‐associated genes in the genome of A. tauschii. Fig. S9 DNA methylase gene expression patterns in the A. tauschii–Bgt incompatible interaction. Fig. S10 Expression patterns of DNA methylase genes upon Bgt inoculation in bread wheat. Fig. S11 Expression patterns of genes with CHH‐hypomethylated differentially methylated regions (DMRs). Fig. S12 Relationship between CHH methylation extent and the expression patterns of various genes after Bgt inoculation in AL8/78. Fig. S13 Phylogenetic relationships of plant DRM2‐related proteins. Fig. S14 Gene structure of DRM3. Fig. S15 Characterization of two DMR genes in response to Bgt infection. Fig. S16 Expression patterns of AeGlu upon Bgt inoculation. Fig. S17 Multiple sequence alignment of endo‐1,3‐beta‐d‐glucosidase genes from wheat‐related species. Fig. S18 Detection of methylation status at the DRM2 gene regions (Fig. 5d) in VIGS plants at 12 hai with Bgt. Fig. S19 Relative transcript levels of AeGlu in mock‐treated (GFP) and DRM2 VIGS AL8/78 plants following Bgt inoculation. Fig. S20 Expression of AePOL IV, AeRDR2, and AeDCL3a in A. tauschii following Bgt inoculation. Table S1 List of primers used in this study. Table S2 Major components of the RNA‐directed DNA methylation (RdDM) pathway in rice and their homologs in A. tauschii and wheat. Table S3 AeAGO4a and AeAGO4b peptides identified by mass spectrometry. Table S4 Statistics of raw small RNA [file NPH-221-1023-s001.pdf]

New Phytologist **Supporting Information Figs S1–S20 and Tables S1–S3 & S6**

Article title: DNA methylation dynamics during the interaction of wheat progenitor *Aegilops tauschii* with the obligate biotrophic fungus *Blumeria graminis* f. sp. *tritici*

Authors: Shuaifeng Geng, Xingchen Kong, Gaoyuan Song, Meiling Jia, Jiantao Guan, Fang Wang, Zhengrui Qin, Liang Wu, Xiujin Lan, Aili Li, and Long Mao
Article acceptance date: 6 August 2018

The following Supporting Information, including Figs S1–S20 and Tables S1–S3 & S6, is available:

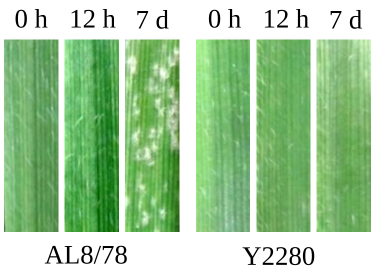

Fig. S1 Response of *Aegilops tauschii* to *Bgt* inoculation. Accession Y2280 was resistant to *Bgt* race no. 15 (virulence type E09) from the Beijing area, while AL8/78 became susceptible by 7 dai. At 12 hai, no clear microcolony formation was observed in either accession.

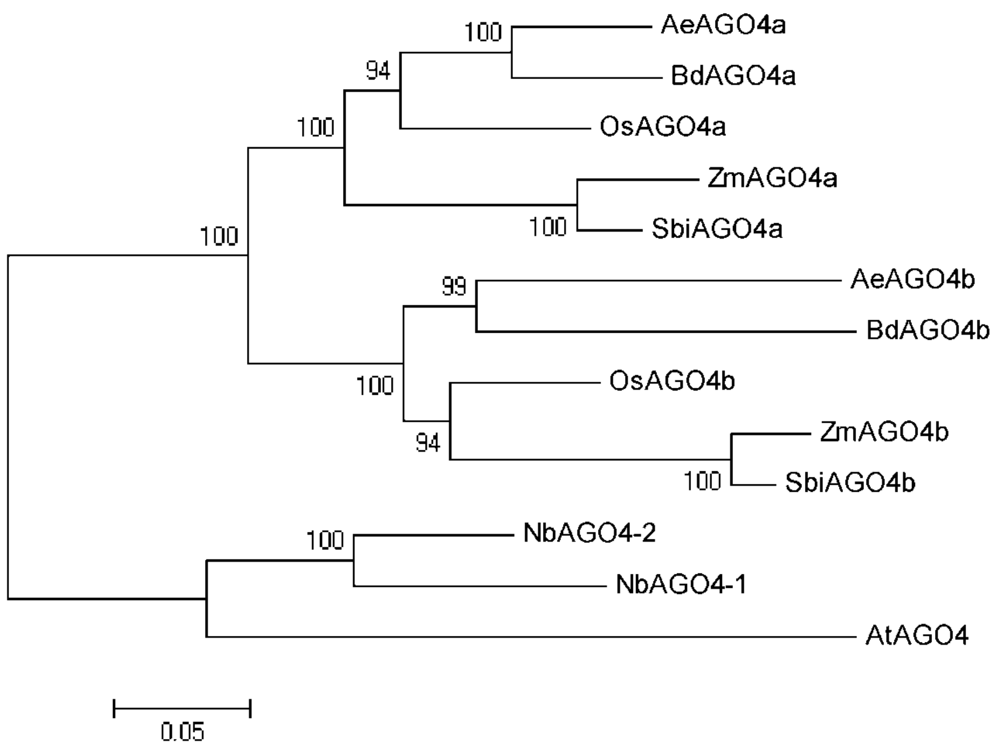

Fig. S2 Phylogenetic relationships of plant AGO4a and AGO4b proteins. Alignments of full-length AGO4 protein sequences were produced by CLUSTALW and used for phylogenetic analysis. The midpoint-rooted phylogenetic tree was constructed with MEGA5 using the Neighbor-Joining method with the equal input substation model and a bootstrap value of 1000 trials. The evolutionary distances were computed as number of amino acid substitutions per site, as shown by the scale below the tree. Species abbreviations: *Aegilops tauschii* (Ae), *Brachypodium distachyon* (Bd), *Zea mays* (Zm), *Sorghum bicolor* (Sbi), *Nicotiana tabacum* (Nb), *Arabidopsis thaliana* (At), and *Oryza sativa* (Os) AGO4 proteins.

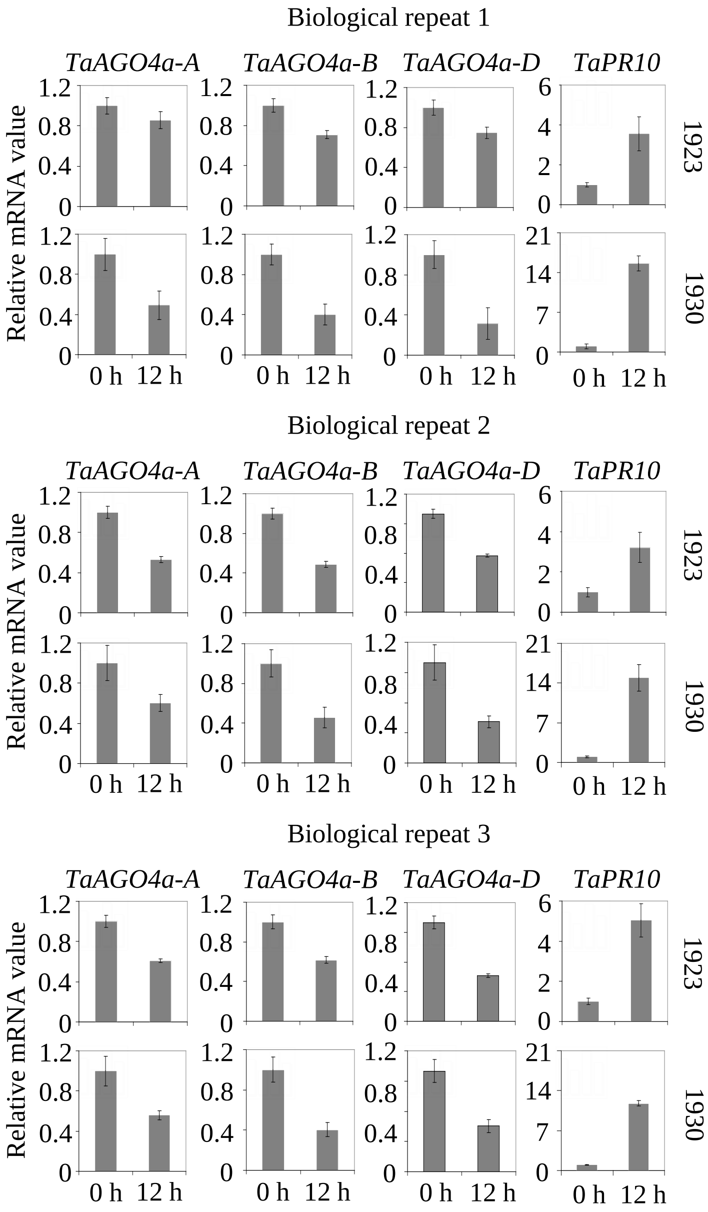

Fig. S3 Expression of *AGO4a* homoeologs and *TaPR10* in bread wheat following *Bgt* inoculation. Leaves from 2-week-old plants of lines 1923 and 1930 were inoculated with *Bgt* for 12 h when RNA was isolated, and mRNA levels of the three homeologs *TaAGO4a-A*, *TaAGO4a-B*, and *TaAGO4a-D* were measured. The pathogenesis-related gene *TaPR10* was used as an indicator of successful inoculation. Data from three biological repeats are shown. Means and SD were calculated with data from three independent biological replicates.

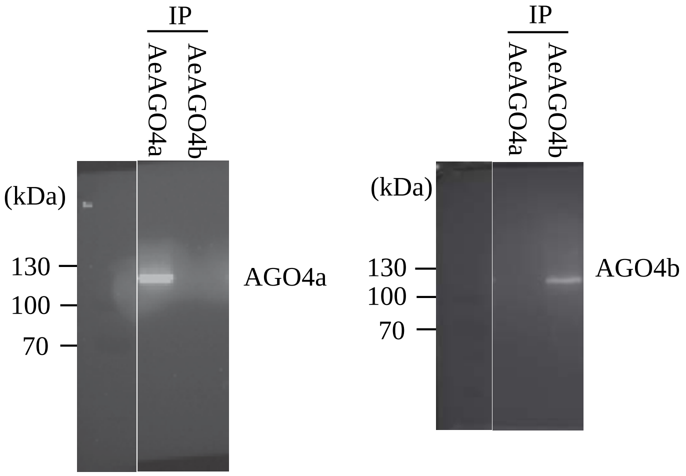

Fig. S4 Immunoblot analysis of AGO4a- and AGO4b-specific antibodies in AL8/78. Leaf samples were collected from 2-week-old AL8/78 plants, and AGO4a and AGO4b were detected using the corresponding antibodies.

(a)

Nominal mass (Mr): 8695; Sequence Coverage: 24%

Matched peptides shown in **Bold Red**

MESHSDDLPPPPPLPPNAEPIKAESADDLPPPPPLPPIKPEEAKKISKPKRALIARPGFGKRG
NPIQLVTNHFKVSLK**TTDEFFHHYYVNLKYEDDRPVDGK**GVGGKVIDKLAQTYPSELAH
KDFAYDGEKSLFTIGALPQINNEFVVVLEDVSSGKTPANGSPGNDSPDKKRVKRPYQTK**TF**
KVELSFAARIPMSAIAMALKGQESEHTQEAIR**VIDIILR**QHSSK**QGCLLVR**QSFFHNNPSNF
VDLGGGVMGCR**GFHSSFR**ATQSGLSLNIDVSTTMIVKPGPVVDFLLANQKVDHPNKIDW
AKAKRALKNLRIKTSPANTEYKIVGLSERNCYEQMFSLKQRNGGNGDPEAIEISVYDYFV
KNRGIELR**YSGDFPCINVGKPRRPTYFPIELCQLVPLQR**YTK**SLSTLQR**SSLVEKSRQKPQE
RMSVLSDVLKRSSYDTEPMLK**ACGISIAQGFTQVSGR**VLQAPKLKAGNGEDIFTRNGRWN
FNNKRLARACVVDRWAVVNFSARCNTMNLVNDLIKCGGMKGITVEKPHIVIEENGSMRR
APAPKRVEDMFEQVKSKLPGAPK**FLLCILAER**KNSDVYGPWKRK**CLADFGIVTQCVAPTR**
VNDQYLTNVLLKINAKLGGMNSLLQIEMSPSIPLVSK**VPTLILGMDVSHGSPGQSDIPSIAA**
VVGSREWPLVSKYRASVRSQSPK**LEMIDSLFKPQGTDDDGLVR**ECLIDFYTSSGKR**KPDQI**
IIFRDGVSESQFNQVLNIELDQIIEACKFLDENWNPKFTLIVAQKNHHTK**FFIPGSPDNVPPG**
TVVDNAVCHPRNYDFYMCAHAGMIGTTRPTHYHILHDEIHFAADDLQDLVHSLSYVYQR
STTAISVVSPICYAHLAAAQVAQFIKFDEMSETSSSQGGGHTSAGSAPVQELPRLHEKVRSS
MFFC

(b)

Nominal mass (Mr): 8469; Sequence Coverage: 9%

Matched peptides shown in **Bold Red**

MDPHDGEPAADELPPPPPLPPNVVPIIAEDAAAAAAGESEPPPPPPPPPSKPAKPRKHIMARP
PNGLGKKGQPIQLLANHYKVSVKPSEEFFNHYYVNLKYEDDTPVDSKGIGRKVLDKLQH
TYHSELANKDFAYDGEKSLFTIGALPQINNEFIVVLEDIGSGKTAAGSPGGNNGSPGGGDQ
KRVRRPYQAKTFKVELNFAATIPMAAIGHAIRGQESEHSLEALRVLDIILRQHSAKQGCLL
VRQSFFHNNPSNFVDLGGGVMGCRGFHSSFRGAQSGLSLNIDVSTTMIVKPGPVIDFLLA
NQKVDHPDKIDWQKAKRALKNLRIKTTPANSEFKIVGLSERNCNEQMFPLRRRNGDSTET
VEITVYDYFVKNRGIELRYSGNLPCINAGRPKRPTYFPVELCTLVPLQRYTKALSTMQRTSL
VEKSRQKPHERMSTLNDALKRSNYDADPMLKACGISIAQNFTQIEGRVLPAPKLKAGNGE
EFFTRNGRWNIARKKLIRTSSVKRWSVVNFSARCDLRGLVQDLK**RVATGMGLEYEDPHTV**
IEESPSLRRAPVARRVEEMFAQIKA**KLPGAPLFLLCLLPERK**NCEVYGPWKKKCLADFGIV
TQCLAPQ**RVNDQYLSNLLLKI**NA**KLGGLNTLLQIEAARA**IPIVGKVPTIILGMDVSHGQPG
QSDRPSIAAVVSSREWPLISKYRATVHTQSPKQEMMASLFKPRGTEDDGLI**RESLIDFYTSS**
GKRKPDQVIIFRDGVSESQFTQVINIELEQIIEACKCLDDKWEPKFTVIVAQKNHHTRFFQA
NSPENVPPGTVVDKQVCHPKNFDFYMCAHAGMIGTSRPTHYHVLHDEIGFSGDELQEFV
HSLSYVYQRSTTAISVAAPIAYAHLAAAQVGTFMKFDDMSDTSSSQGGGHTSAGSAPVPE
LPRLHEKVRSSMFFC

Fig. S5 Mass spectrometry analyses of purified *A. tauschii* AGO4 proteins. (a) AGO4a; (b) AGO4b. Matched peptides shown in Bold Red.

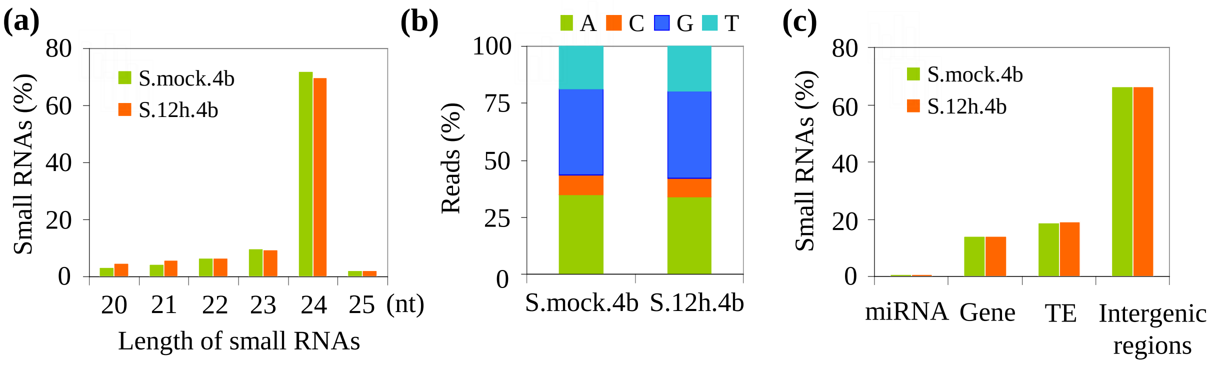

Fig. S6 Characterization of 24-nt sRNAs pulled down by AGO4b in *A. tauschii*. (a) Size distribution of sequenced sRNAs pulled down by AGO4b in AL8/78. (b) Relative frequency of each first nucleotide of sRNAs pulled down by AGO4b in AL8/78. (c) Distribution of 24-nt small RNAs pulled down by AGO4b in miRNA, gene, TE, and intergenic regions.

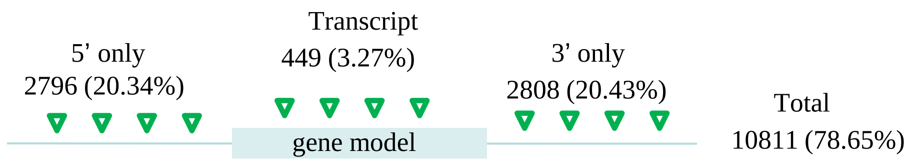

Fig. S7 Proportions of TAGs and locations of TEs (triangles) in *A. tauschii* gene models. Transcribed regions, light blue box; 2-kb upstream and downstream regions, extended lines.

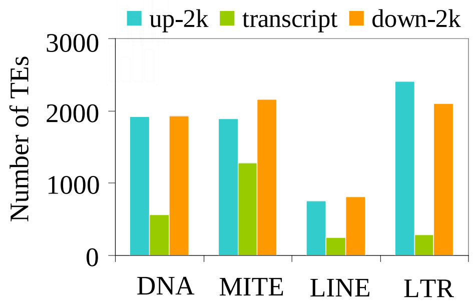

Fig. S8 Distribution of TEs relative to genic regions of TE-associated genes in the genome of *A. tauschii*. Transcript, ORF; up-2k, 2 kb upstream of the ORF; down-2k, 2 kb downstream of the ORF.

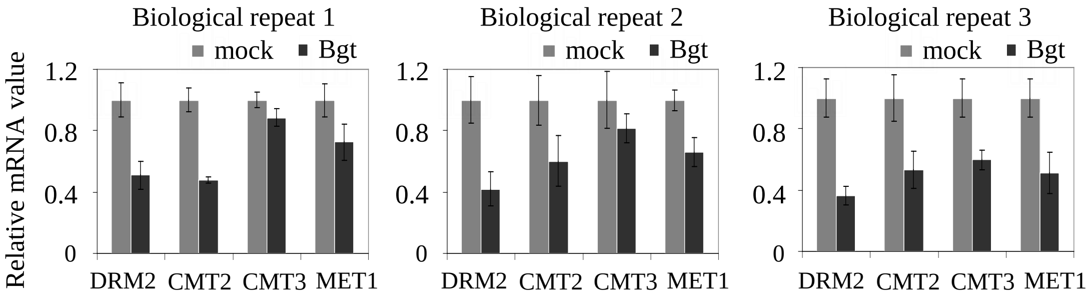

Fig. S9 DNA methylase gene expression patterns in the *A. tauschii*–*Bgt* incompatible interaction. RNA analyzed was from leaves of the 2-week-old line Y2280 12 hai with *Bgt*. Data from three biological repeats are shown. Means and SD were calculated for data from three independent biological replicates.

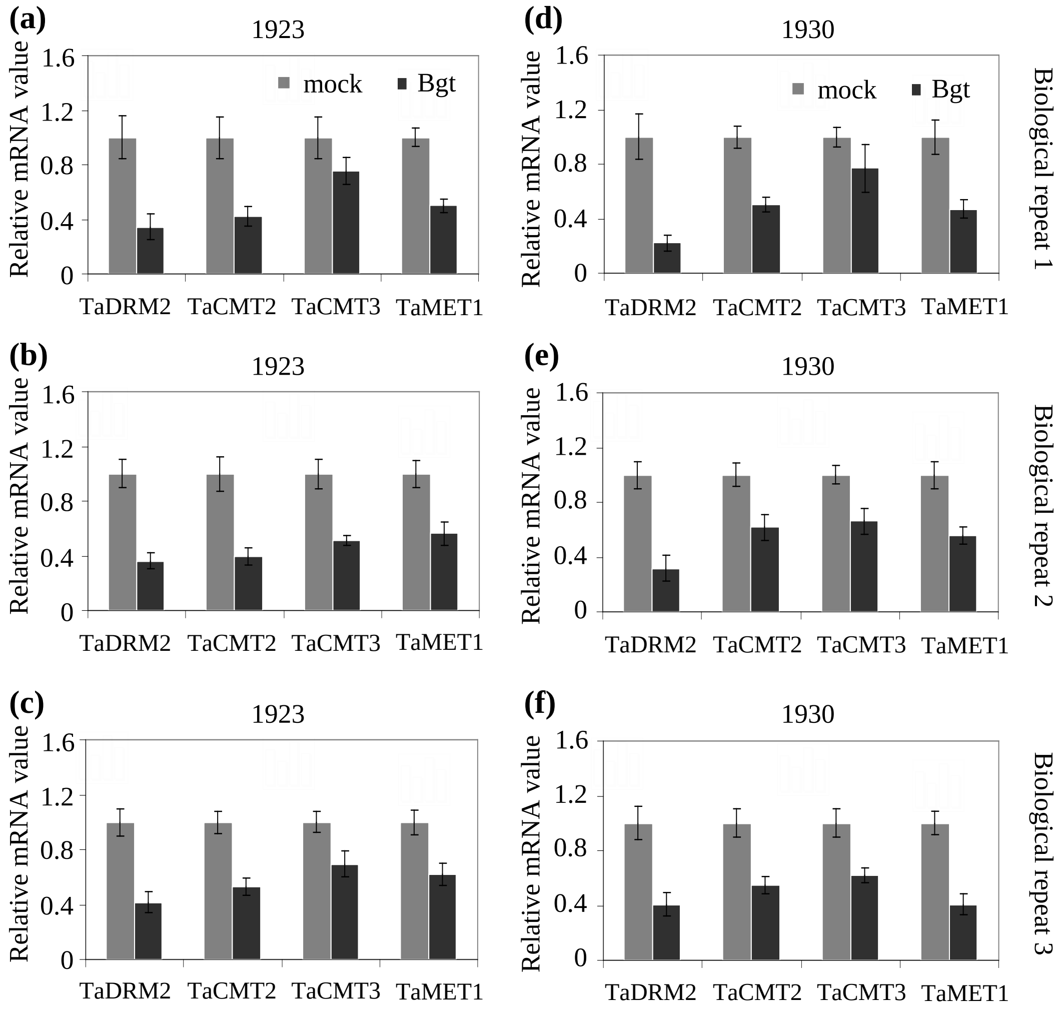

Fig. S10 Expression patterns of DNA methylase genes upon *Bgt* inoculation. *Bgt*-susceptible line PmAm6/Beijing837 BC5F3 1923 (a-c) and *Bgt*-resistant line 1930 (d-f) following a compatible interaction with *Bgt* at 12 hai. Data from three biological repeats are shown individually. Means and SD were calculated with data from three independent biological replicates.

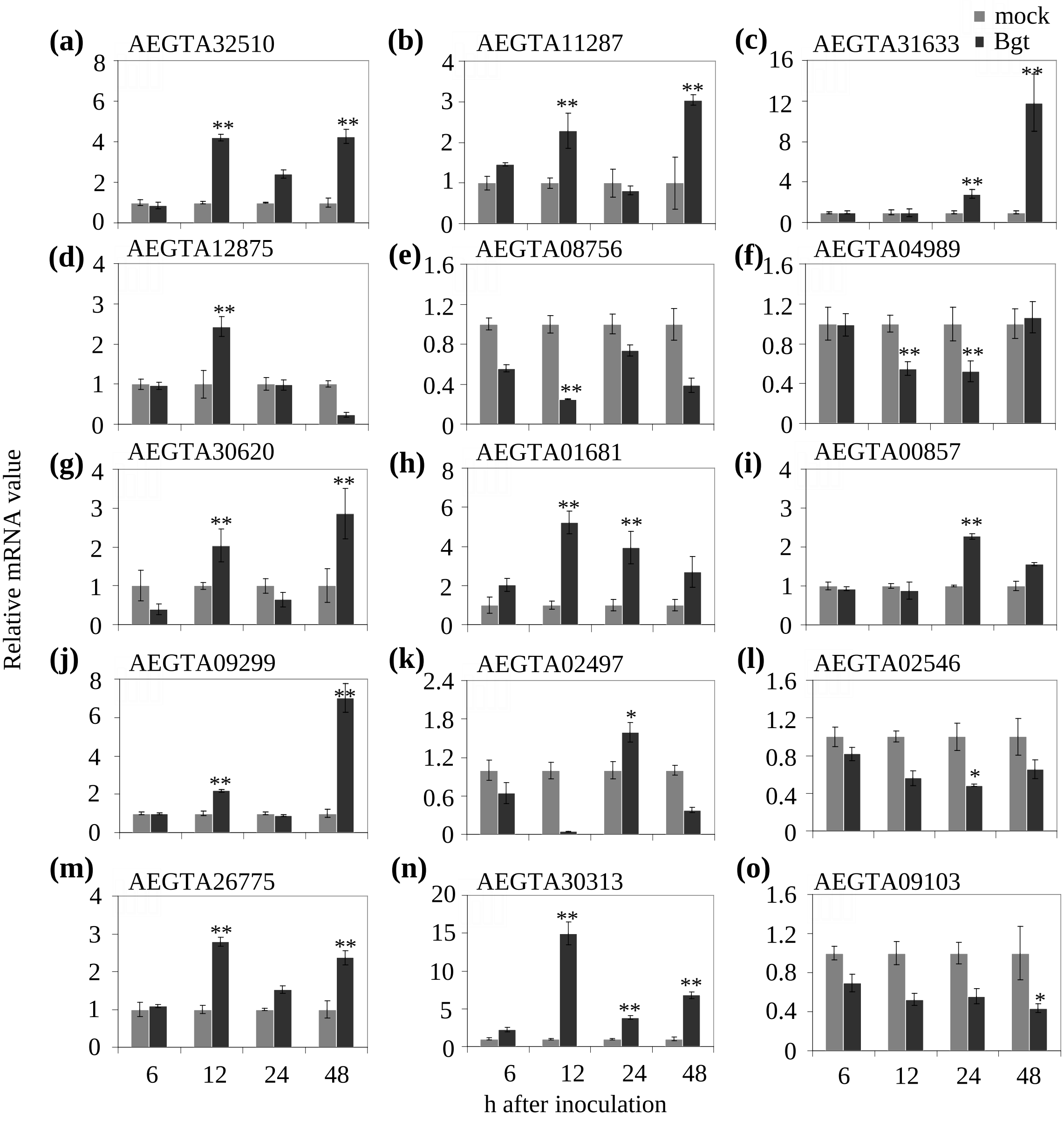

Fig. S11 Expression patterns of genes with CHH-hypomethylated differentially methylated regions (DMRs). The genes were selected from the MapMan biotic stress bin. (a–f) Genes with promoter-localized DMRs and TE-associated genes (TAG). (g–l) Genes with promoter-localized DMRs only. (j–l) Genes with downstream DMRs and TE (TAG). (m–o) Genes with downstream DMRs only. For RT-PCR, *GAPDH* was used as an internal reference. RNA samples were collected from AL8/78 plants at 6, 12, 24, and 48 hai. AEGTA32510, abiotic stress gene encoding fatty acid alpha-dioxygenase; AEGTA11287, secondary metabolism gene encoding phenylpropanoid; AEGTA31633, AEGTA12875, and AEGTA02546, signalling.receptor kinases; AEGTA08756, AEGTA04989, and AEGTA02497, stress.biotic.PR-proteins; AEGTA30620, abiotic stress gene; AEGTA01681, RNA regulation, WRKY-related transcription factor; AEGTA00857, cell wall; AEGTA09299, signaling gene of phytosulfokine receptor precursor; AEGTA26775, glutathione S transferase gene; AEGTA30313, peroxidase gene; and AEGTA09103, RNA regulation, MYB-related transcription factor. Error bars indicate SD of three independent experiments. Student's *t*-test, **P*<0.05, ***P*<0.01.

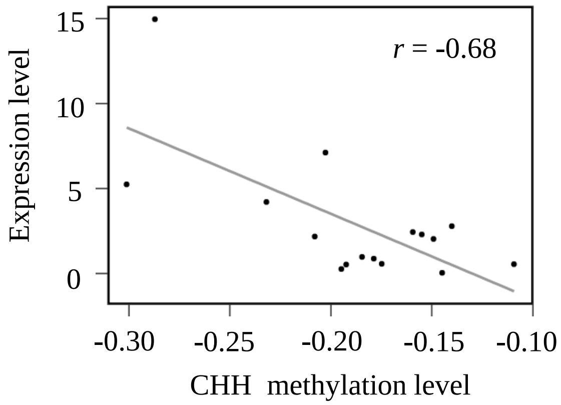

Fig. S12 Relationship between CHH methylation extent and the expression patterns of various genes after *Bgt* inoculation in AL8/78.

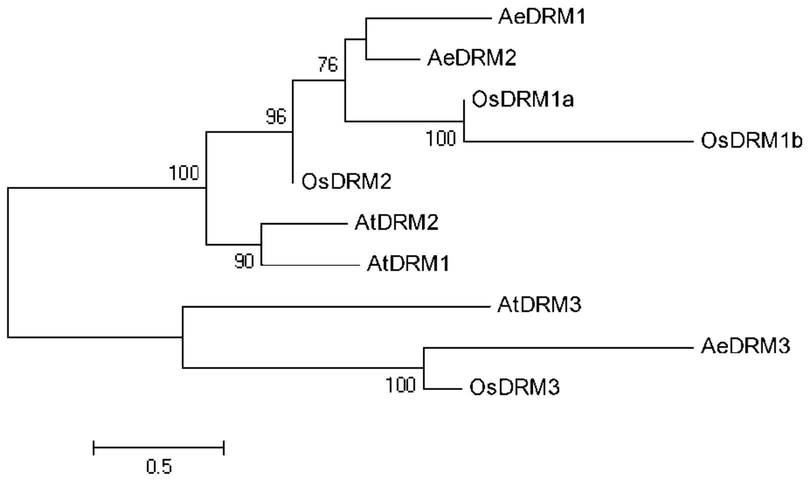

Fig. S13 Phylogenetic relationships of plant DRM2-related proteins. Alignments of full-length DRM protein sequences were produced by CLUSTALW and subjected to phylogenetic analysis. The midpoint-rooted phylogenetic tree was constructed using MEGA5, and the equal input substitution model with bootstrap values from 1000 trials was used for Neighbor-Joining. The evolutionary distances were computed in number of amino acid substitutions per site, as shown by the scale below the tree. *Aegilops tauschii* (Ae), *Arabidopsis thaliana* (At), and *Oryza sativa* (Os).

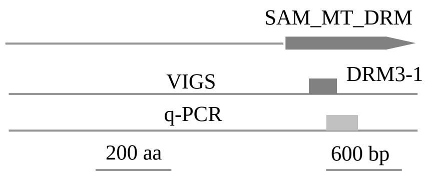

Fig. S14 Gene structure of *DRM3*. Structure was predicted by ScanProsite (http://prosite.expasy.org/scanprosite/). DRM3-1: boxes indicate DNA fragments used for VIGS construct development, and the regions used for qPCR are indicated by gray boxes.

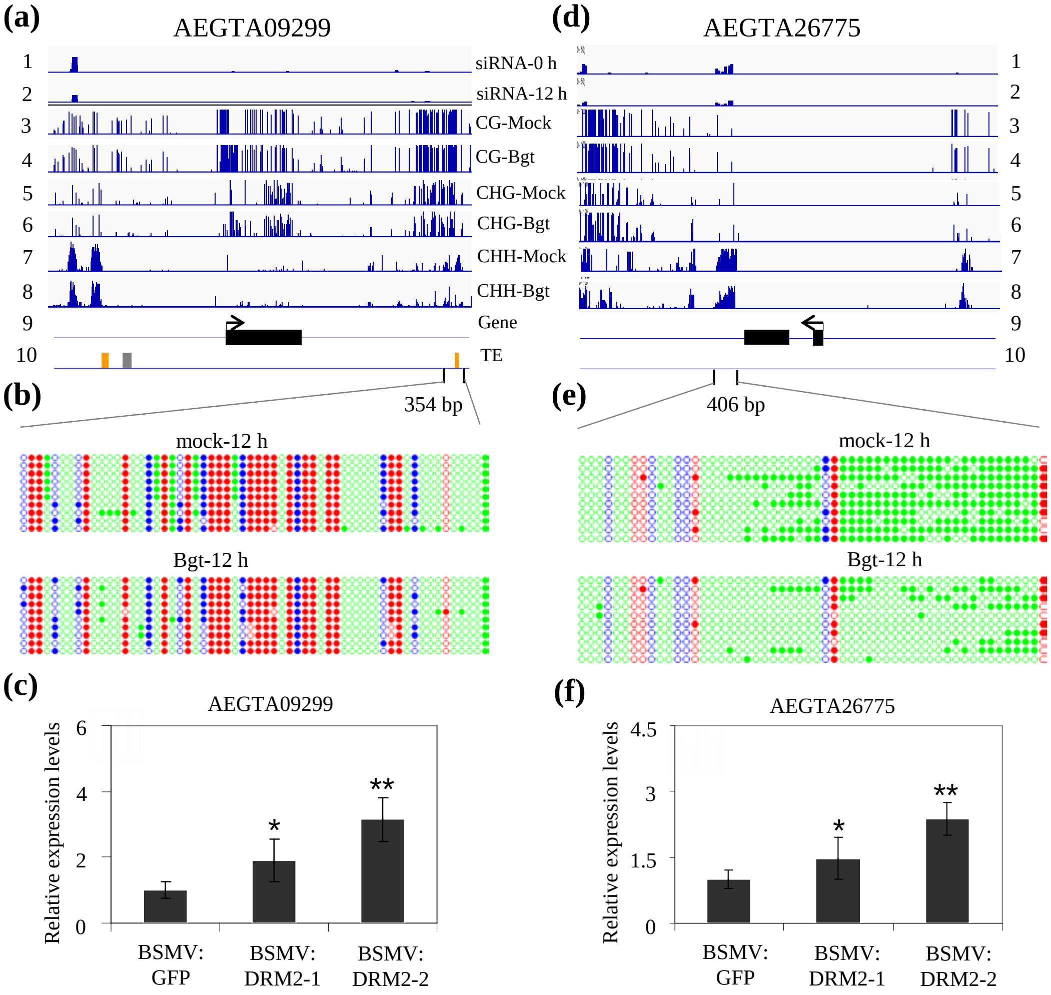

Fig. S15 Characterization of two DMR genes in response to *Bgt* infection. (a) Gene structure of AEGTA09299, encoding a phytosulfokine receptor for signaling (line 9, black boxes represent exons) and its associated TEs (line 10; yellow boxes, TcMar-Stowaway; gray box, DNA). The lines above the gene models show the distribution and density of 24-nt small RNAs (lines 1 and 2), CpG methylation (lines 3 and 4), CHG methylation (lines 5 and 6), and CHH methylation (lines 7 and 8) in mock-treated and AL8/78 leaves at 12 hai with *Bgt*. (b) Confirmation of differential CHH methylation at the MITE region by PCR amplification and sequencing of bisulfite-treated DNA. Colors represent different methylation contexts: CG (red), CHG (blue), and CHH (green). Cytosines are indicated with empty circles when un-methylated and filled circles when methylated. (c) Relative transcript levels of AEGTA09299 in control and *DRM2* VIGS plants. (d–f) analysis of AEGTA26775, a glutathione S transferase gene for redox. Error bars indicate SD of three independent experiments. Student's *t*-test, *P<0.05, **P<0.01.

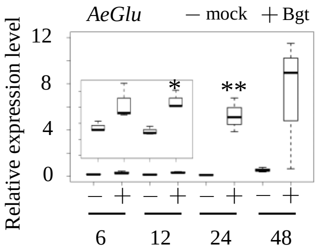

Fig. S16 Expression patterns of *AeGlu* upon *Bgt* inoculation. Student's *t*-test, **P*<0.05, ***P*<0.01.

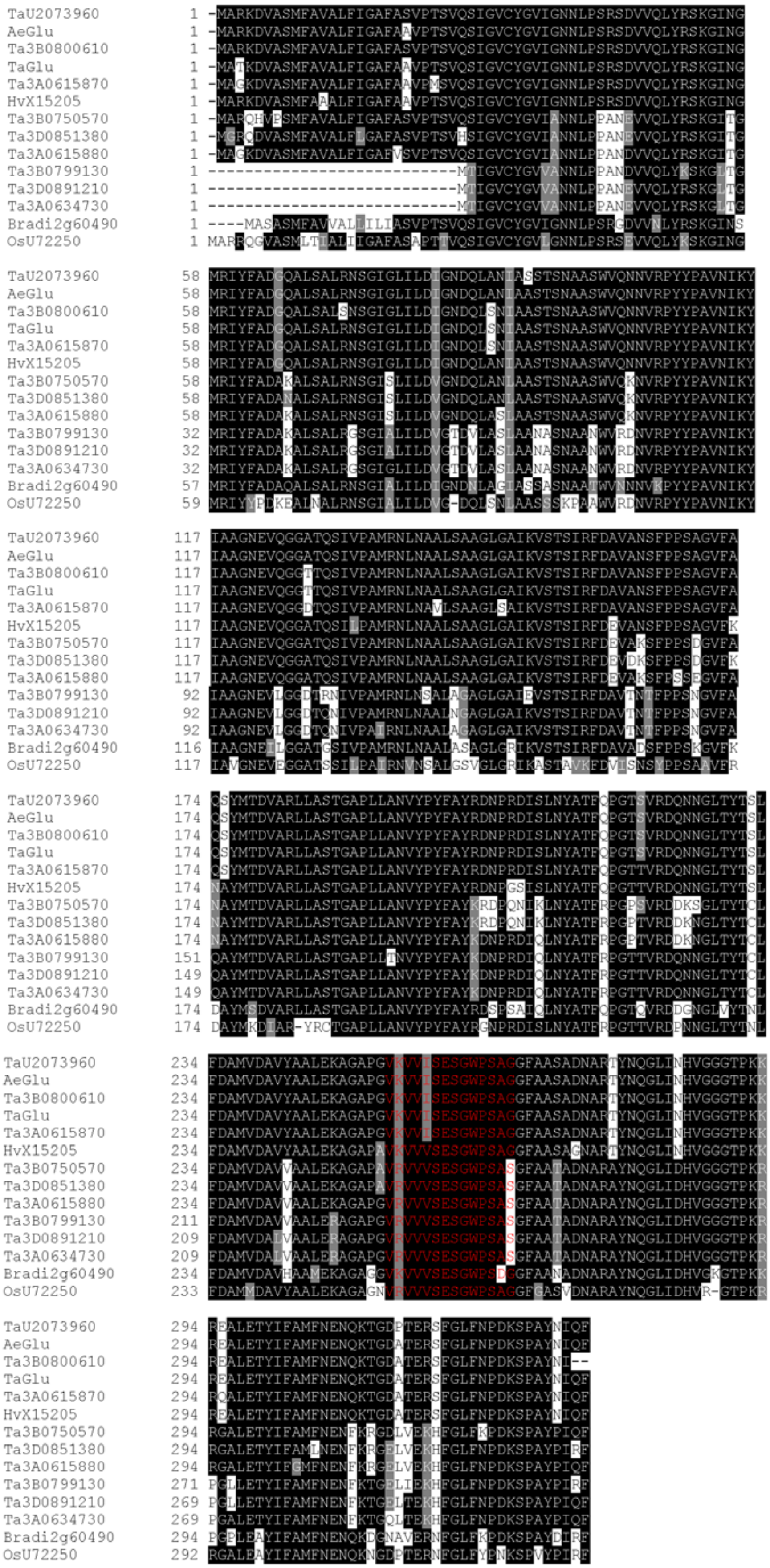

Fig. S17 Multiple sequence alignment of endo-1,3-beta-D-glucosidase genes from wheat-related species. Red residues indicate glycosyl hydrolases family 17 signature; Black shading residues conserved in all alignment sequence. Sequence names and their annotations: AeGlu, AEGTA30224; TaGlu, Y18212; TaU2073960, Triticum_aestivum_TRIAE_CS42_U_TGACv1_640781_AA2073960; Ta3B0800610, Triticum_aestivum_TRIAE_CS42_3B_TGACv1_224720_AA0800610; Ta3A0615870, Triticum_aestivum_TRIAE_CS42_3AL_TGACv1_193635_AA0615870; Ta3B0750570, Triticum_aestivum_TRIAE_CS42_3B_TGACv1_221825_AA0750570; Ta3D0851380, Triticum_aestivum_TRIAE_CS42_3DL_TGACv1_249546_AA0851380; Ta3A0615880, Triticum_aestivum_TRIAE_CS42_3AL_TGACv1_193635_AA0615880; Ta3B0799130, Triticum_aestivum_TRIAE_CS42_3B_TGACv1_224635_AA0799130; Ta3D0891210, Triticum_aestivum_TRIAE_CS42_3DL_TGACv1_252640_AA0891210; Ta3A0634730, Triticum_aestivum_TRIAE_CS42_3AL_TGACv1_194515_AA0634730; barley, HvX15205; *Brachypodium*, Bradi2g60490; and rice, OsU72250 (GenBank: U72250).

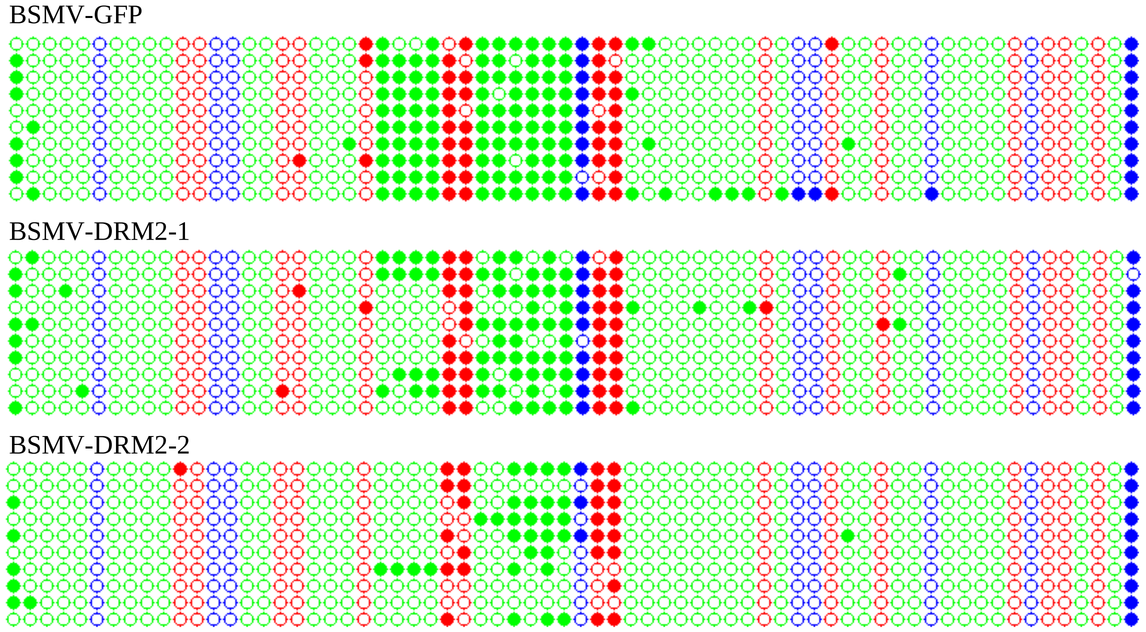

Fig. S18 Detection of methylation status at the *DRM2* gene regions (Fig. 5d) in VIGS plants at 12 hai with *Bgt*. Colors represent different methylation contexts: CG (red), CHG (blue), and CHH (green). Cytosines are indicated by empty circles when un-methylated and filled circles when methylated. BSMV:GFP, GFP as a non-target control; BSMV:DRM2-1 and BSMV:DRM2-2, two DRM2 VIGS lines.

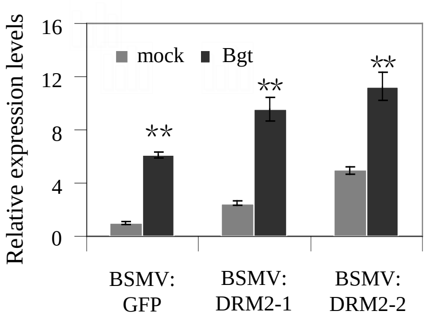

Fig. S19 Relative transcript levels of *AeGlu* in mock-treated (GFP) and DRM2 VIGS AL8/78 plants following *Bgt* inoculation. Leaf segments 3–4 cm long were collected from 6 to 10 fourth leaves of GFP and DRM2 VIGS AL8/78 plants after *Bgt* inoculation. Leaves from at least 15 plants were tested for each vector. Error bars represent the SD among at least three independent replicates. Student's *t*-test, ***P*<0.01.

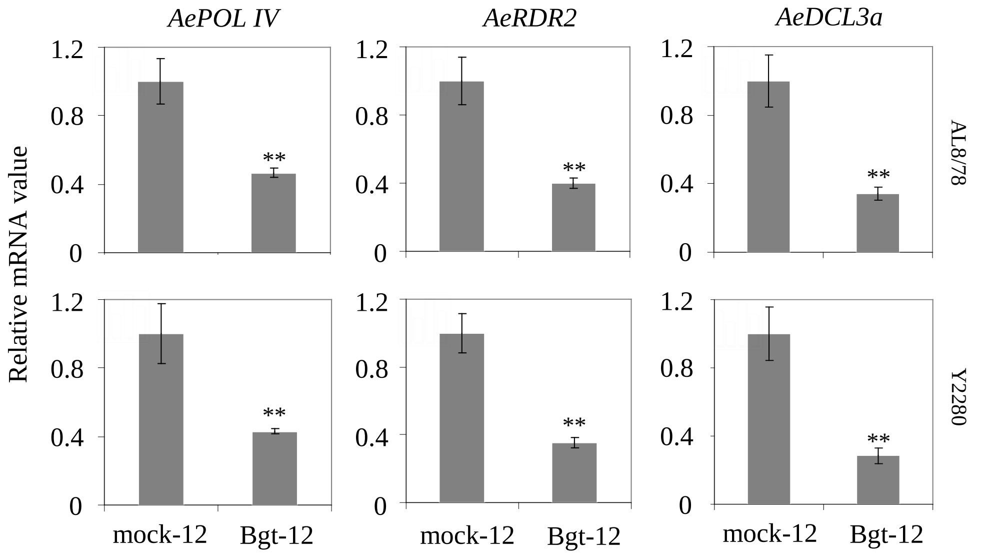

Fig. S20 Expression of *AePOL IV*, *AeRDR2*, and *AeDCL3a* in *A. tauschii* following *Bgt* inoculation. The leaves of 2-week-old AL8/78 and Y2280 plants were inoculated with *Bgt,* and the mRNA levels of these genes were measured at 12 hai. Error bars indicate SD of three independent experiments. Student's *t*-test, ***P*<0.01.

Table S1 List of primers used in this study

Primer name	Forward primer sequences（5’-3’）	Reverse primer sequences（5’-3’）
AeAGO4a	CAGATGGAGTCACACAGTGAT	CTTTGAGATCTTCTTTGCTTC
AeAGO4b	GAGTTCAAGATTGTTGGGTT	ACAGTTTCGGTGCTGTCA
AePR5	ACCAACCGCTGCTCCTT	TCCACGTTTCGCCTCTG
TaAGO4a-A	CAGATGGAGTCACACGGAGAG	TAGGCTTTGAGATCTTCTTTG
TaAGO4a-B	GATGGAGTCGCAAGGTGA	GCTTCTTCAGGTTTGATAGGC
TaAGO4a-D	CAGATGGAGTCACACAGTGAT	CTTTGAGATCTTCTTTGCTTC
TaPR10	GAAACAATTTGCCAGCAG	AGACCACCTTCCACATCC
DRM2	CACCACCCACTTCGTTAG	ATCACTGTGCCCTGAAAG
CMT2	CCGCAACGAAACTACCGA	GCCATAACCCTTCCAACG
CMT3	GGAAAACGAGGCTGATTA	AGTACCAACGGCAGGTGA
MET1	GGATACCAGGTTCGTTTT	CAGGCAGTGTTATTTTCAGT
AeGlu	CGCTGCTGTTCCTACGAGTG	ATGCCGTTGATGCCCTTG
AEGTA32510	CCTGTTCCCATCGGTTTA	GGTGGTCGTTGTACTTGC
AEGTA11287	CTGCCAGGAGAACCAGGAC	ACCAAAGGAGGGCAAAGC
AEGTA09299	CGGATAATTCATTGGTTGG	TGTTCGTCGAGTGTTCTTCTA
AEGTA09103	CACGACCGAAGCGTAAAG	GCGACAACTGGTGGGATA
AEGTA30620	ATGTCGCCTCAGTCTTTC	GTTAGCATCTGGGGTTTT
AEGTA26775	CACCCGATGTTGACAAACG	CCCGTACTTGCGGAGGAT
AEGTA30313	TGAGGCTACCCACCTACAACC	GGACGGAACCATCGCAAC
AEGTA01681	ACATCGCCTTCCAACAAT	TCCAGGGCTCAACATCAG
AEGTA31633	GGTAAGGCTTTCACTAACAT	AGATACAGTATTGCGGCTC
AEGTA12875	CTAAATGGAACGCTACAA	CAAGCAAAGCCAAAGAT
AEGTA08756	GCACAAGAATGCCTCA	ACTCCGCAGATTAGCA
AEGTA04989	TTCTCGTCGTATGTCGG	GCATTGCTGGTGCTAAA
AEGTA00857	CGAGGAACTGTTGAAGGA	GCTGACCACGCAAGGATT
AEGTA02497	CCCTGCTGATAACTCTG	CTGTTACTGTTAGCCACTC
AEGTA02546	GACTGCCTCCCTTGACT	TCCTGTAACTGCCGATT
AePOL IV	GGCGGAGTTGCCCTAT	CGGACCGTTTGCTGAG
AeRDR2	AAATCCGTTCAGTATCTAAGT	GAAATCTTCCCAATACCA
AeDCL3a	TATTTACTACGGATGTCGC	GGGAAGAAACCAGGGA
GAPDH	TTAGACTTGCGAAGCCAGCA	AAATGCCCTTGAGGTTTCCC
DRM3	TGTGAGATGTTGGGAAAG	AAAAGATAAGCAATGGTGT
DRM2-1	GCTAGCGTTTGAGTGGGAAAGTGA	GCTAGCCTAACGAAGTGGGTGGT
DRM2-2	GCTAGCCCTTTGCCCTCCTGTTT	GCTAGCTTGTCTCCATCCCAGTT
DRM3-1	GCTAGCTTACTTCAGAGGTGGCA	GCTAGCTGTATGGTTTGATGGGT
AeGlu-B	AAYAATGGYTAGGAAGGATGTTG	TTCTCTTCCCTRCATTCTTTCTTTT
Ae09299-B	ATATTGATTTAAGGATTYAGTGG	CACATTTTTCACATRTACATATTCT
Ae26775-B	AYGTATYYAAGATGTAGTAATATAT	TAATTTRTRCACTARTACTCCCTCC
BSMV:CP	TACTGTATTTACCTTCGCAT	CTTTCAAGCTTAGCCATTT
Tubulin	AGAACACTGTTGTAAGGCTCAAC	GAGCTTTACTGCCTCGAACATGG

Table S2 Major components of the RNA-directed DNA methylation (RdDM) pathway in rice and their homologs in *A. Tauschii* and wheat

	DD id	CS id	E-Value	Os id	E-Value
Pol IV-dependent siRNA biogenesis					
AGO4a	AEGTA27134	Traes_3AS_8EE711E2C.1	0	LOC_Os01g16870	0
		Traes_3B_F4E4667F8.1	0		
		Traes_3DS_57EA31670.1	0		
AGO4b	AEGTA27714	Traes_1DL_64B330BBB.1	0	LOC_Os04g06770	0
		Traes_1AL_095416BC0.1	0		
		Traes_1BL_7C037D478.1	0		
POL IV	AEGTA07056	Traes_1BS_B0BB95B97.1	0	LOC_Os04g48370	0
		Traes_1AS_3EF7D1A61.1	0		
		Traes_1DS_FA7FC5C3C.1	e-119		
RDR2	AEGTA11614	Traes_2DL_6DB81005E.1	0	LOC_Os04g39160	0
		Traes_2BL_A723D3322.1	0		
		Traes_2AL_9834B38EB.1	0		
DCL3a	AEGTA05560	Traes_3DL_2DC78B18A.1	0	LOC_Os01g68120	0
		Traes_3AL_562D6614F.1	0		
		Traes_3B_2C82C2B7B.1	0		
Pol V-mediated de novo methylation					
DRM2	AEGTA29765	Traes_5AL_BA3074F36.1	0	LOC_Os03g02010	0.00E+00
		Traes_5BL_169D5FB79.1	0		
		Traes_5DL_3A965D7A6.1	0		
Methylation					
CMT2	AEGTA22602	Traes_4AS_7A955D191.1	0	LOC_Os05g13790	3.00E-40
		Traes_4DL_61FDD4D19.1	0		
		Traes_4BL_B35C9BF58.1	0		
CMT3	AEGTA03864	Traes_6DL_7626A87FF.1	0	LOC_Os10g01570	e-164
		Traes_6BL_64DEE7723.1	0		
		Traes_6AL_9E0046317.1	0		
MET1	AEGTA01434	Traes_2BS_173E86712.1	0	LOC_Os07g08500	0.00E+00
		Traes_2AS_9B3DEDDAB.1	0		
		Traes_2DS_D3601814B.1	0		
**Other**					
DRM3	AEGTA20109	Traes_5DS_DE9039E5C.1	0	LOC_Os05g04330	e-161
		Traes_5AS_BBB41162C.1	0		
		Traes_5BS_B90CCD62B.1	0		
PR5	AEGTA29062	Traes_4AL_1F77AED931.1	3.00E-59	LOC_Os12g43430	6.00E-62
		Traes_5AS_381655EE4.1	1.00E-58		
		Traes_2AS_84C021B0B.1	6.00E-58		
PR10	AEGTA07257	Traes_5DS_8F312CB951.1	3.00E-74	LOC_Os12g36860	1.00E-34
		Traes_5BS_91BD3E004.1	6.00E-71		
		Traes_5AS_FAD05211F.1	5.00E-06		

Table S3 AeAGO4a and AeAGO4b peptides identified by mass spectrometry

Purified	start	end	length（AA）
AGO4a	79	102	24
	182	192	11
	216	222	7
	228	234	7
	257	263	7
	373	403	31
	407	413	7
	448	463	16
	569	577	9
	590	621	32
	643	672	30
	691	710	20
	724	732	9
	779	802	24
AGO4b	530	554	25
	571	587	17
	614	627	14
	630	645	16
	719	732	14

Table S6 Summary of cytosine methylation status in mock- and *Bgt*-inoculated samples

Sample	mock-inoculation	Bgt-inoculated
Total Sequence pairs of high quality reads analyzed	1222056977	824077864
Number of paired-end alignments with a unique best hit	629528945	428932138
Mapping efficiency	51.50%	52.00%
Bisulfite conversion efficiency %	99.95%	99.92%
Total number of C's analyzed	24073447013	17487400832
% C's in CpG context that are methylated	87.1	86.44
% C's in CHG context that are methylated	59.35	57.64
% C's in CHH context that are methylated	1.92	1.64
